# Forsythoside A Mitigates Alzheimer's-like Pathology by Inhibiting Ferroptosis-mediated Neuroinflammation via Nrf2/GPX4 Axis Activation

**DOI:** 10.7150/ijbs.69714

**Published:** 2022-02-28

**Authors:** Chunyue Wang, Shanshan Chen, Hangyu Guo, Hongbo Jiang, Honghan Liu, Haoran Fu, Di Wang

**Affiliations:** 1 School of Life Sciences, Jilin University, Changchun, 130012, China.; 2 Engineering Research Center of Chinese Ministry of Education for Edible and Medicinal Fungi, Jilin Agricultural University, Changchun, 130118, China.

**Keywords:** Alzheimer's disease, forsythoside A, neuroprotection, neuroinflammation, ferroptosis, Nrf2/GPX4 axis

## Abstract

Ferroptosis and neuroinflammation play crucial roles in Alzheimer's disease (AD) pathophysiology. Forsythoside A (FA), the main constituent of *Forsythia suspensa* (Thunb.) Vahl., possesses anti-inflammatory, antibacterial, antioxidant, and neuroprotective properties. The present study aimed to investigate the potential role of FA in AD neuropathology using male APP/PS1 double transgenic AD mice, Aβ_1-42_-exposed N2a cells, erastin-stimulated HT22 cells, and LPS-induced BV2 cells. FA treatment significantly improved mitochondrial function and inhibited lipid peroxidation in Aβ_1-42_-exposed N2a cells. In LPS-stimulated BV2 cells, FA treatment decreased the formation of the pro-inflammatory factors IL-6, IL-1β, and NO. In male APP/PS1 mice, FA treatment ameliorated memory and cognitive impairments and suppressed Aβ deposition and p-tau levels in the brain. Analyses using proteomics, immunohistochemistry, ELISA, and western blot revealed that FA treatment significantly augmented dopaminergic signaling, inhibited iron deposition and lipid peroxidation, prevented the activation of IKK/IκB/NF-κB signaling, reduced the secretion of pro-inflammatory factors, and promoted the production of anti-inflammatory factors in the brain. FA treatment exerted anti-ferroptosis and anti-neuroinflammatory effects in erastin-stimulated HT22 cells, and the Nrf2/GPX4 axis played a key role in these effects. Collectively, these results demonstrate the protective effects of FA and highlight its therapeutic potential as a drug component for AD treatment.

## Introduction

Alzheimer's disease (AD) is the most prevalent neurodegenerative disorder causing dementia 1. The pathophysiological processes underscoring AD are thought to begin 20 years or more prior to the onset of cognitive symptoms 2, which negatively impact patients' health and place a substantial economic burden on individuals, families, and society 3. In 2019, the oligosaccharide GV-971 was approved for the treatment of mild and moderate AD in China and was assessed in an international phase III clinical trial in April 2020 4. In June 2021, the U.S. Food and Drug Administration approved aducanumab, co-developed by Biogen and Eisai, for the treatment of AD 5. Despite these encouraging findings, the pathological mechanisms underlying AD remain unclear, which precludes the development of more effective treatments 6.

Iron deposits are observed in plaques, microglia, and tangle-bearing neurons in the AD brain 7. Iron is a key component of ferroptosis, which is defined as the accumulation of lethal lipid substances produced by lipid peroxidation. Ferroptosis has emerged as a major process in AD pathophysiology 8 and has become a target for AD treatment. Previous studies have demonstrated that almost all genes related to ferroptosis are regulated by nuclear factor erythroid 2-related factor 2 (Nrf2) transcription, including, but not limited to, nicotinamide adenine dinucleotide phosphate, glutathione peroxidase 4 (GPX4), and iron-regulated genes 8, 9. As a member of the selenoprotein glutathione peroxidase family, GPX4 has the unique function of reducing hydroperoxide in membrane lipids 10. This property of reducing phospholipid hydroperoxide, thus inhibiting lipoxygenase-mediated lipid peroxidation, underscores its vital role in preventing ferroptosis 8. Nevertheless, the role of Nrf2/GPX4 in AD remains poorly understood.

In addition to ferroptosis, brain tissue is particularly vulnerable to neuroinflammation, which can aggravate amyloid-β (Aβ) deposition and tau phosphorylation 11. Ferroptosis is associated with neuroinflammation, suggesting that iron retention can trigger microglial activation and interleukin (IL)-1β production in AD 7. Additionally, the Nrf2 pathway inhibits reactive oxygen species (ROS)-related nuclear factor-κB (NF-ĸB) activation by preventing the degradation of inhibitor of NF-κB (IĸB)-α 12. As a downstream target of Nrf2, GPX4 prevents tumor necrosis factor (TNF)-mediated activation of NF-ĸB. Correspondingly, neuroinflammation is suppressed via inhibition of ferroptosis 13. Although ferroptosis inhibitors are considered as key regulators of neuroinflammation, the role of Nrf2/GPX4 in the regulation of the NF-ĸB signaling pathway in AD has yet to be elucidated.

**Figure 1:**
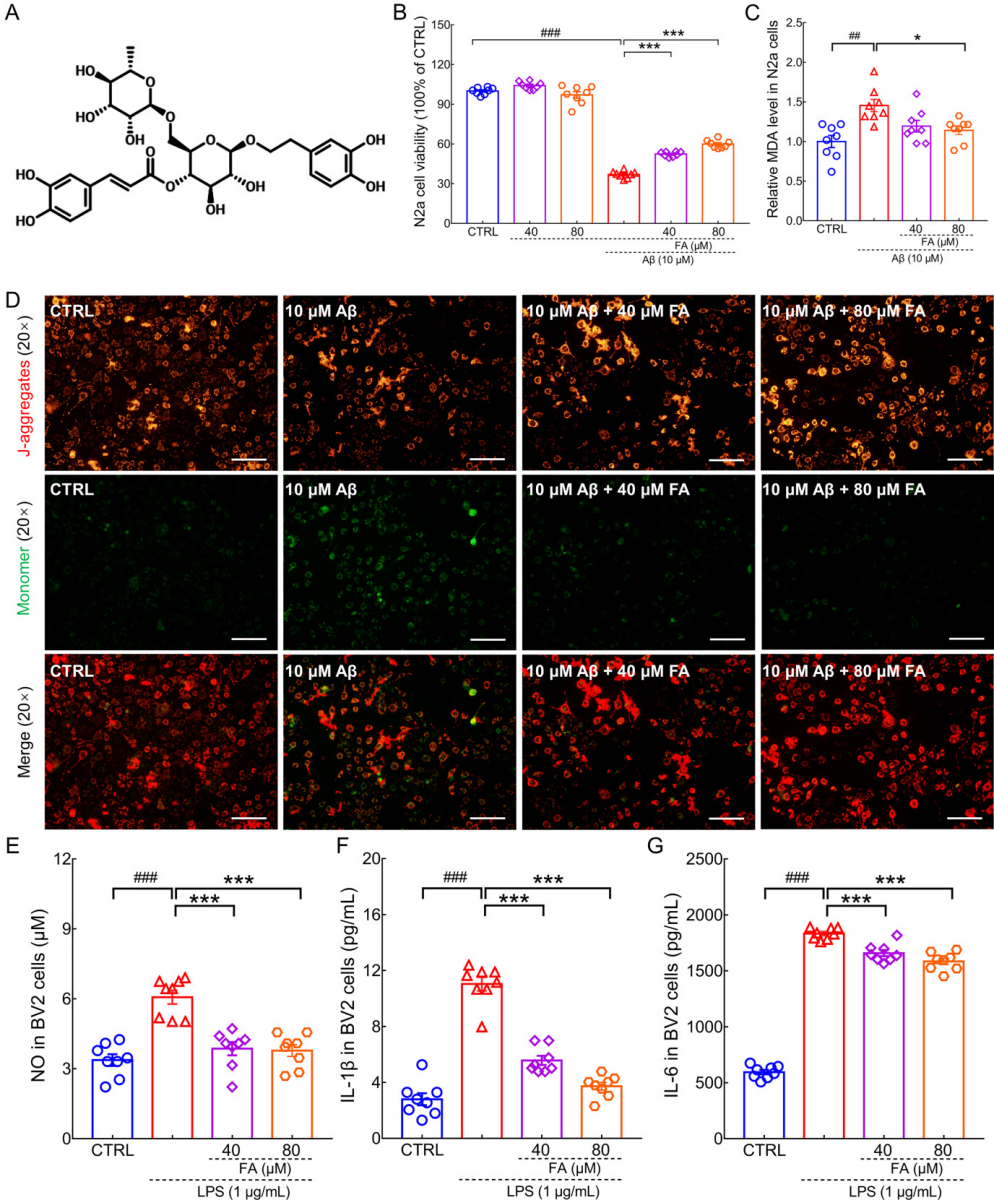
**FA treatment protects N2a cells and BV2 cells against Aβ_1-42_ and LPS toxicity.** (**A**) Chemical structure of FA (CAS: 79916-77-1). **(B)** FA treatment enhanced cell viability in Aβ_1-42_-exposed N2a cells without influencing cell viability alone (*n* = 8). (**C**) FA treatment downregulated MDA levels in Aβ_1-42_-exposed N2a cells (*n* = 8). (**D**) FA treatment prevented the dissipation of MMP in Aβ_1-42_-exposed N2a cells (*n* = 3). Scale bar: 100 µm. Red and green fluorescence signals represent J-aggregates and monomers, respectively. FA treatment decreased the concentration of **(E)** NO, **(F)** IL-1β, and **(G)** IL-6 in LPS-exposed BV2 cells (*n* = 8). The data are presented as mean ± S.E.M. ^##^*p* < 0.01, and ^###^*p* < 0.001 vs. CTRL N2a cells for **(B)** and **(C)**; ^###^*p* < 0.001 vs. CTRL BV2 cells for **(E), (F),** and **(G)**; *p < 0.05, and ***p < 0.001 vs. Aβ_1-42_-stimulated N2a cells for **(B)** and **(C)**; ***p < 0.001 vs. LPS-exposed BV2 cells for **(E), (F),** and **(G).**

Forsythoside A (FA) (3,4-dihydroxy-β-phenethyl-O-α-L-rhamnopyranosyl-(1→6)-4-O-caffeoyl-β-D-glucopyranoside) (Fig. 1A), also referred to as forsythiaside, is the main component of *Forsythia suspensa* (Thunb.) Vahl (*F. suspensa* or Lianqiao in Chinese), which was first recorded in Shen Nong's Herbology, the earliest extant treatise in Chinese pharmacological literature 14. FA possesses various beneficial pharmacological characteristics, including anti-inflammatory, anti-oxidative, and neuroprotective properties 15. Oral administration of FA was reported to decrease the number of microglia and astrocytes in transient cerebral global ischemia in gerbils 16. Further, FA treatment attenuated neuroinflammation and apoptosis caused by Aβ in hippocampal slices 17, improved memory and learning abilities in senescence-accelerated mouse-prone 8 mice 15, and reduced Aβ_25-35_-induced apoptosis in PC12 cells 18. However, the effects of FA treatment on ferroptosis in AD have not been reported to date.

In this study, we explored the effects of FA treatment on AD neuropathology in male APP/PS1 double transgenic AD mice, Aβ_1-42_-exposed N2a cells, erastin-stimulated HT22 cells, and lipopolysaccharide (LPS)-induced BV2 cells in order to identify its mechanism of action.

## Materials and methods

### Animals and ethical statement

**Figure 2:**
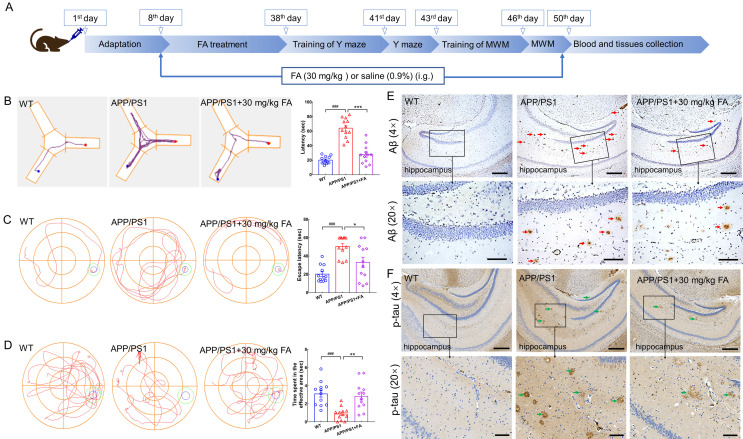
** FA treatment ameliorates AD symptoms in APP/PS1 mice.** (**A**) Schematic diagram of animal experiments. (**B**) FA treatment shortened foraging time of APP/PS1 mice in the Y-maze test (*n* = 12). (**C**) FA treatment shortened the escape latency of APP/PS1 mice in the MWM navigation test (*n* = 12). (**D**) FA treatment increased the time of APP/PS1 mice spent in the effective area in the MWM probe trials (no platform) (*n* = 12). The blue and green circles indicate the position of the original platform and effective area, respectively. (**E**) FA treatment decreased Aβ_1-42_ (red arrow) deposition in the hippocampus of APP/PS1 mice (*n* = 3). (**F**) FA treatment suppressed the levels of phosphorylated tau protein (green arrow) in the hippocampus of APP/PS1 mice (*n* = 3). Scale bars: 400 µm for 4× magnification and 100 µm for 20× magnification. The data are presented as mean ± S.E.M. ^###^*p* < 0.001 vs. WT mice; *p < 0.05, **p < 0.01 and ***p < 0.001 vs. APP/PS1 mice.

All animal experiments were approved by the Animal Ethics Committee of Jilin University (permit No. SY201905013) and were conducted in compliance with the ARRIVE guidelines. Eight-month-old B6C3-Tg (APPswePSEN1dE9)/Nju double transgenic male mice (APP/PS1) (genotype: (Appswe) T, (Psen1) T) and age-matched wild-type (WT) (genotype: (Appswe) W, (Psen1) W) male mice were purchased from Nanjing Biomedical Research Institute of Nanjing University. All mice were individually housed at 24 °C with food and drinking water available *ad libitum*. After 1 week of adaption in the new environment, WT mice received oral administration of normal saline (10 mL/kg) and were designated as the control group (*n* = 12). APP/PS1 mice were randomly divided into two groups: the model group (*n* = 12) received oral administration of normal saline (10 mL/kg) and the agent-treated group (*n* = 12) received oral treatment with 30 mg/kg FA (L-012-171216, 98.83% purity, Chengdu Herbpurify Co., Ltd., Chengdu, China) beginning on day 8. After 30-day treatment, behavioral experiments were serially performed. The entire treatment protocol lasted for 42 days. Blood samples were collected from the caudal vein. After euthanasia via CO_2_ inhalation, organs including the brain, liver, spleen, and kidney were collected for further analysis (Fig. 2A).

### Behavioral tests

#### Y-maze test

Working memory was evaluated using the Y-maze test. The Y-maze comprised three arms (dimensions of each arm: 30 cm × 6 cm × 30 cm), including one start arm and two objective arms. After 3-day training, the mice were fasted for 12 h prior to the formal test. On day 41, the mice were individually placed at the end of the start arm, and food was placed in one of the two objective arms. The movement trajectory of each mouse and time spent searching for food were recorded with a camera and analyzed using Any-maze software (Stoelting Co., Chicago, IL, USA) under the condition of researchers blinded to treatment groups.

#### Morris water maze (MWM) test

Spatial memory and navigation ability were evaluated using the MWM test on day 43. Both training and formal testing were performed using an MT-200 water labyrinth video-tracking analysis system (MT-200, TECHMAN Software Co., Ltd., Chengdu, China). The cistern was divided into four quadrants. Titanium dioxide was added to the cistern to make the water turbid. During training, the mice were placed in the water to reach the platform within 60 s. After finding the platform, the mice stayed on the platform for 20 s. The mice that did not find the platform within 60 s were manually led to the platform and stayed there for 20 s. After 3 days of training, the navigation test and probe trial were performed under blinded conditions on days 46 and 47, respectively. As reported in our previous study 19, the mice were placed into the cistern from the opposite side of the platform, and the time it took for mice to find the platform and/or the time they passed through the effective area were recorded.

### Pathology assessments

Immunohistochemistry assays were conducted as reported previously 20. Briefly, 4% paraformaldehyde-fixed brain slides were deparaffinized, and then antigen retrieval was performed with a citric acid antigen retrieval buffer (pH 6.0) (G1202, servicebio technology CO., LTD, Wuhan, China) in a microwave oven. After incubating with primary antibodies (Table S1) at 4 °C overnight, the sections were incubated with biotin-conjugated AffiniPure goat anti-rabbit IgG (H+L) (Table S1). Images were obtained using an optical microscope (BX51, Olympus, Tokyo, Japan).

### Label-free quantification proteomics

Label-free quantification proteomics was performed as previously described 19. Briefly, 100 mg of mouse hippocampal tissue was collected with 1,000 μL ice-cold radio immunoprecipitation assay (RIPA) (20-188, Sigma-Aldrich, St. Louis, MO, USA) containing 1% proteolytic protease and phosphatase inhibitor cocktail (P002, New Cell & Molecular Biotech Co., Ltd., Suzhou, China), which were added for tissue homogenization at 4 °C. After centrifugation at 4 °C for 15 min, proteins were precipitated using acetone. The samples were then resuspended and mixed with trypsin, incubated overnight at 37 °C, and then desalted after the removal of sodium deoxycholate. A volume of 2 μL of polypeptide was analyzed using nano ultra-performance liquid chromatography (EASY-nLC1200) coupled with Q-Exactive mass spectrometry (Thermo Finnigan). MaxQuant (Version 1.5.6.0) was used to analyze the raw MS files. The protein sequence database was obtained from UniProt (Uniprot_mouse_2016_09). Significantly differentially expressed proteins were defined as proteins with a content ratio between the two groups (WT group/APP/PS1 group or FA-treated APP/PS1 group/APP/PS1 group) of > 1.5 or < 0.66. These differentially expressed proteins were subjected to cluster analysis, protein interaction analysis, gene ontology (GO) analysis, and Kyoto Encyclopedia of Genes and Genomes (KEGG) analysis.

### Cellular experiments

#### Cell cultures

HT22 cells derived from a mouse hippocampal neuronal cell line (CL-0595, Procell Life Science & Technology Co., Ltd., Wuhan, China) were cultured in Dulbecco's modified Eagle's medium (C11995500BT, Thermo Fisher Biochemical Products Co., Ltd., Waltham, MA, USA) containing 10% fetal bovine serum (164210, Procell Life Science & Technology Co., Ltd.), and 1% penicillin-streptomycin solution (15140, Thermo Fisher Biochemical Products Co., Ltd.). Mouse neuroblastoma N2a cells (CX0020, Boster Biological Technology Co., Ltd., Wuhan, China) and BV2 microglial cells (CL-0493, Procell Life Science & Technology Co., Ltd.) were maintained in minimum essential medium (PM150410, Procell Life Science & Technology Co., Ltd.) supplemented with 10% fetal bovine serum and 1% penicillin-streptomycin solution. Cells were cultured in a humidified atmosphere with 5% CO_2_ at 37 °C.

#### Cell viability assay

HT22 cells were seeded in a 96-well plate (5 × 10^3^ cells per well) and pretreated with FA (40 μM and 80 μM) for 3 h, followed by 24-h co-exposure to 10 μM of erastin (HY-15763, MedChemExpress, Shanghai, China). N2a cells (5 × 10^3^ cells per well) were seeded in a 96-well plate and pretreated with FA (40 μM and 80 μM) for 3 h, followed by 24-h co-exposure to 10 μM of Aβ oligomers (052487, GL Biochem Ltd., Shanghai, China). Cell viability was measured using 3-(4,5-dimethylthiazolyl-2-yl)-2,5-diphenyl tetrazolium bromide (MTT) (S19063, Shanghai Yuanye Bio-Technology Co., Ltd., Shanghai, China) at 490 nm, as described in our previous study 20.

### Mitochondrial membrane potential (MMP) detection

MMP was evaluated using 5,5′,6,6′-Tetrachloro-1,1′,3,3′-tetraethyl-imidacarbocyanine iodide (JC-1) staining (C2006, Beyotime, Shanghai, China) according to the manufacturer's instructions. N2a cells (2 × 10^5^ cells per well) were seeded in a 6-well plate and incubated overnight. Cells were pretreated with FA (40 μM and 80 μM) for 3 h followed by stimulation with 10 μM of Aβ oligomers. After 24-h co-incubation, cells were incubated with 500 μL of JC-1 at 37 °C for 30 min in the dark. Cells were washed in phosphate buffer solution (PBS), and fluorescence images were subsequently obtained using a fluorescence microscope (TH4-200, Olympus Corporation, Japan).

### Glutathione (GSH) assay

HT22 cells were pretreated with 40 μM and 80 μM of FA for 3 h, followed by co-incubation with 10 μM of erastin for another 24 h. GSH content in HT22 cells was evaluated according to the manufacturer's instructions (S0053, Beyotime, Shanghai, China).

### Lipid ROS detection

HT22 cells (2 × 10^5^ cells per well) were seeded in a 6-well plate and pretreated with FA (40 μM and 80 μM) for 3 h, followed by 24-h co-exposure to 10 μM of erastin. Cells were incubated with BODIPY 581/591 C11 (D3861, Thermo Fisher Biochemical Products Co., Ltd., Waltham, MA, USA) at a final concentration of 10 μM for 30 min in the dark. After three washes in PBS, fluorescence images were obtained using a fluorescence microscope (TH4-200, Olympus Corporation, Japan).

### Griess assay

The amount of nitric oxide (NO) produced by BV2 cells is positively related to nitrite release, which is considered an indicator of NO generation. BV2 cells were pretreated with FA (40 μM and 80 μM) for 3 h, followed by 24-h co-exposure to 1 μg/mL of LPS (DH183-1, Beijing Dingguo Changsheng Biotechnology Co., Ltd., Beijing, China). A volume of 50 μL of supernatant was collected and added to a fresh 96-well plate containing an equivalent volume of Griess reagent I and Griess reagent II (S0021, Beyotime, Shanghai, China). The absorbance was measured at 540 nm using a microwave reader (HBS-1096A, Detie, Nanjing, China).

### Transmission electron microscopy (TEM)

As described in our previous study 19. HT22 cells were pretreated with FA (40 μM and 80 μM) for 3 h, followed by co-incubation with 10 μM of erastin for 24 h. After fixation with 2.5% glutaraldehyde at 4 °C for 4 h, the cells were fixed with 1% osmic acid-0.1 M PBS (pH 7.4) for 2 h at 25 °C, dehydrated in an ethanol gradient, permeabilized, and polymerized at 60 °C for 48 h. Ultrathin sections of 60-80 nm were prepared and stained with a saturated aqueous solution of 2% uranyl acetate and lead citrate. The ultrastructure of HT22 cells was observed using TEM (TECNAI G2 20 TWIN, FEI, Hillsboro, OR, USA).

### RNA interference (RNAi) of *GPX4* and *Nrf2*

HT22 cells were transfected with small interfering RNA (siRNA) targeting *GPX4* (50 nM) (Q1208, RiboBio, Guangzhou, China) and *Nrf2* (50 nM) (U0805, RiboBio, Guangzhou, China) to knock down the expression of *GPX4* and *Nrf2*, respectively. Negative control (NC) siRNA (50 nM) (S1012, RiboBio, Guangzhou, China) served as the control group. After 24 h of transfection, HT22 cells were treated with FA or erastin and then used for subsequent experiments. All transfections were performed using LipoRNAi transfection reagent (C0535, Beyotime, Shanghai, China).

### Malondialdehyde (MDA) assay

Normal or *GPX4*/*Nrf2* siRNA-transfected HT22 cells were pretreated with FA (40 μM and 80 μM) for 3 h, followed by co-incubation with 10 μM of erastin for 24 h. Normal N2a cells were pretreated with FA (40 μM and 80 μM) for 3 h, followed by co-incubation with 10 μM of Aβ oligomers for 24 h. All treated cells were collected, and the protein concentration was determined using a BCA Protein Assay Kit (23225, Thermo Fisher Scientific, Waltham, MA, USA) according to the manufacturer's instructions. MDA production was analyzed using a cell MDA assay kit (A003-4-1, Nanjing Jiancheng Bioengineering Institute, Nanjing, China) according to the manufacturer's instructions.

### Enzyme-linked immunosorbent assay (ELISA)

Brain samples were collected from APP/PS1 mice and were lysed using PBS buffer. Protein concentration was determined using a BCA Protein Assay Kit. The levels of transforming growth factor-β (TGF-β) (RK00057), monocyte chemoattractant protein-1 (MCP-1) (RK00381), IL-1β (RK00006), IL-6 (RK00008), and TNF-α (RK00027) in brains were analyzed using a commercial kit (ABclonal, Wuhan, China) according to the manufacturer's instructions. BV2 cells were pretreated with FA (40 μM and 80 μM) for 3 h, followed by co-incubation with 1 μg/mL of LPS for 24 h. The levels of IL-1β (EK0394) and IL-6 (EK0411) in the collected culture medium were analyzed using a commercial kit (Boster, Wuhan, China) according to the manufacturer's instructions.

### Western blot

Normal or *GPX4*/*Nrf2* siRNA-transfected HT22 cells were pretreated with FA (40 μM and 80 μM) for 3 h, followed by co-incubation with 10 μM of erastin for 24 h. Brain samples were collected from APP/PS1 mice, and the collected cells were lysed using ice-cold RIPA containing 1% lytic protease and phosphatase inhibitor cocktail. Protein concentration was determined using a BCA Protein Assay Kit. Protein samples were separated by sodium dodecyl sulfate-polyacrylamide gel electrophoresis and transferred to polyvinylidene fluoride membranes (0.45 μm) (10600023, Cytiva, Breisgau, Germany). Membranes were blocked with a highly efficient commercial blocking solution (GF1815, Genefist, Culham Science Centre, Oxfordshire, UK) for 15 min, followed by overnight incubation with primary antibodies (Table S1) at 4 ºC and 2-h incubation with horseradish peroxidase-conjugated secondary antibodies (Table S1) at 25 ºC. The protein bands were detected using an ultra-sensitive electrochemiluminescence kit (P2300, New Cell & Molecular Biotech Co., Ltd., Suzhou, China) with an imaging system (Tanon 5200, Tanon Science & Technology Co., Ltd., Shanghai, China) and analyzed using ImageJ 6.0 software (National Institutes of Health, Bethesda, Maryland, USA).

### Statistical analysis

All data are presented as mean ± S.E.M. BONC DSS Statistics 25 software (Business-intelligence of Oriental Nations Corporation Ltd., Beijing, China) was used for statistical analysis, and GraphPad Prism 9 software (GraphPad Software Inc., San Diego, CA, USA) was used for generating graphs. A one-way analysis of variance (ANOVA) followed by Tukey's post hoc test was performed. Statistical significance was set at *p* < 0.05.

## Results

### FA treatment protected cells against Aβ_1-42_ and LPS-induced injury

Aβ is thought to promote neuronal death by damaging mitochondria, aggravating neuroinflammation and oxidative stress 21. Inflammation of the central nervous system is closely associated with neurodegenerative diseases. Microglia own an important position and number in neuronal cell populations in the brain region and are activated during neuroinflammation 22. In this study, the classic preclinical AD models of Aβ**_1-42_**-induced N2a cells and LPS-induced BV2 microglia were used to explore the neuroprotective effects of FA. FA treatment dose-dependently improved cell viability (*p* < 0.001) (Fig. 1B), decreased MDA levels (*p* < 0.05) (Fig. 1C), and prevented the dissipation of MMP (*p* < 0.01) (Fig. 1D and Fig. S1) in Aβ_1-42_-exposed N2a cells, suggesting that FA protects against Aβ_1-42_ toxicity. LPS has been reported to induce inflammation in microglia 23. In LPS-stimulated BV2 cells, FA treatment dose-dependently reduced NO (*p* < 0.001) (Fig. 1E), IL-1β (*p* < 0.001) (Fig. 1F), and IL-6 (*p* < 0.001) (Fig. 1G) production, indicative of anti-inflammatory properties.

### FA treatment ameliorated AD-like symptoms in APP/PS1 mice

Six-week FA administration suppressed the high index levels in the liver (*p* < 0.001) (Fig. S2B) and kidneys (*p* < 0.01) (Fig. S2D) of APP/PS1 mice, without influencing their body weight (Fig. S2A) and spleen index (Fig. S2C). No evident pathological changes were observed in the liver (Fig. S3A), spleen (Fig. S3B), and kidney (Fig. S3C) after FA administration, supporting the safety of FA treatment in mice.

In the Y-maze test, the foraging time of APP/PS1 mice was longer than that of WT mice (*p* < 0.001). In contrast, foraging time was shortened after FA administration in APP/PS1 mice (*p* < 0.001) (Fig. 2B). Memory and spatial recognition abilities of mice were assessed using the MWM. In APP/PS1 mice, FA treatment shortened the escape latency in the navigation test (*p* < 0.05) (Fig. 2C) and increased the time spent in the effective area in the probe trials (no platform) (*p* < 0.01) (Fig. 2D).

Aβ deposition and neurofibrillary tangles formed by highly phosphorylated tau protein are considered the two major pathological hallmarks of AD 19. Compared with vehicle treatment, FA treatment strongly suppressed Aβ deposition (Fig. 2E) and high p-tau levels (Fig. 2F) in the hippocampus. These data suggested that FA treatment ameliorated AD-like symptoms in APP/PS1 mice.

### FA treatment augmented dopaminergic signaling and prevented ferroptosis in APP/PS1 mice

**Figure 3:**
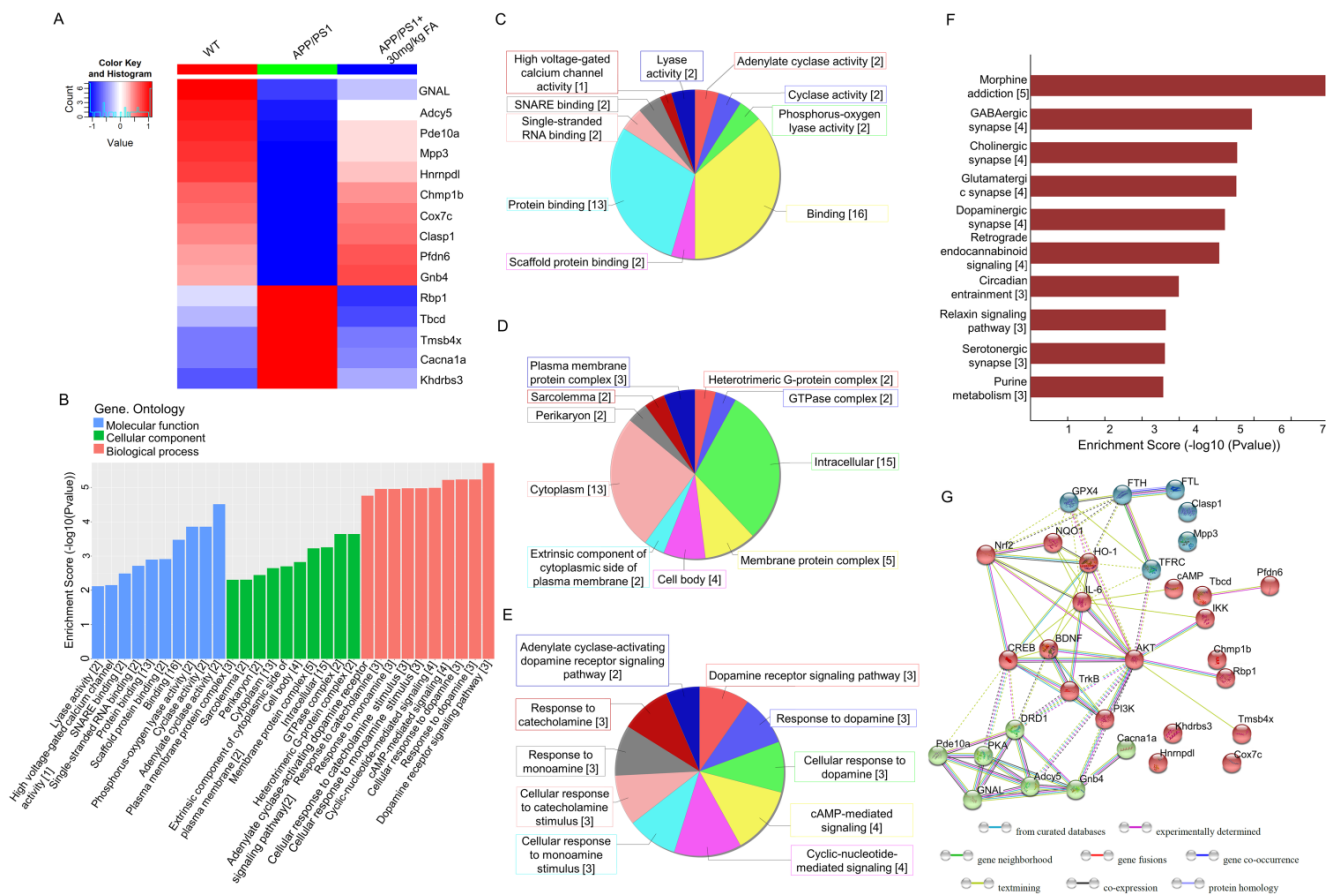
**Proteomics and bioinformatics analysis of the hippocampus in APP/PS1 mice**. (**A**) Heat map of 15 proteins. Red and blue indicate high-abundance and low-abundance proteins, respectively. (**B**) GO enrichment analysis of differentially expressed proteins among WT, APP/PS1, and FA-treated APP/PS1 mice. Molecular function, cellular component, and biological process are marked in blue, green, and pink, respectively. (**C**) Pie chart of GO molecular function classification. (**D**) Pie chart of GO cellular component classification. (**E**) Pie chart of GO biological process classification. (**F**) KEGG enrichment analysis of differentially expressed proteins among WT, APP/PS1, and FA-treated APP/PS1 mice. Rows represent individual biological processes and the number of related altered proteins. (**G**) Protein-protein interaction network analysis generated using STRING software.

Label-free proteomics was performed to screen proteins that were significantly differentially expressed among WT, APP/PS1, and FA-treated APP/PS1 mice. The analysis identified 15 proteins with significantly different expression (Fig. 3A and Table S2), among which, adenylate cyclase 5 (Adcy5) and guanine nucleotide-binding protein G(olf) subunit α (GNAL) were associated with cyclic adenosine monophosphate (cAMP) production and dopaminergic downstream signaling pathways 24, 25. GO analysis detected cyclase activity, cAMP-mediated signaling, and dopamine (DA) receptor signaling pathways (Fig. 3B-E). KEGG analysis revealed that significantly differentially expressed proteins were associated with dopaminergic synapses (Fig. 3F). As a key neurotransmitter in the nervous system, DA prevents ferroptosis by reducing iron accumulation and degrading GPX4 in cells 26. DA receptors inhibit NF-κB activity via the protein phosphatase 2A-dependent protein kinase B (AKT) pathway, thereby inhibiting inflammation 27. Based on biosynthesis, the relationships among the 15 significant proteins, ferroptosis-related proteins, and neuroinflammation-related proteins are presented in the STRING interaction analysis (Fig. 3G), implying the regulation of ferroptosis and neuroinflammation by FA.

**Figure 4:**
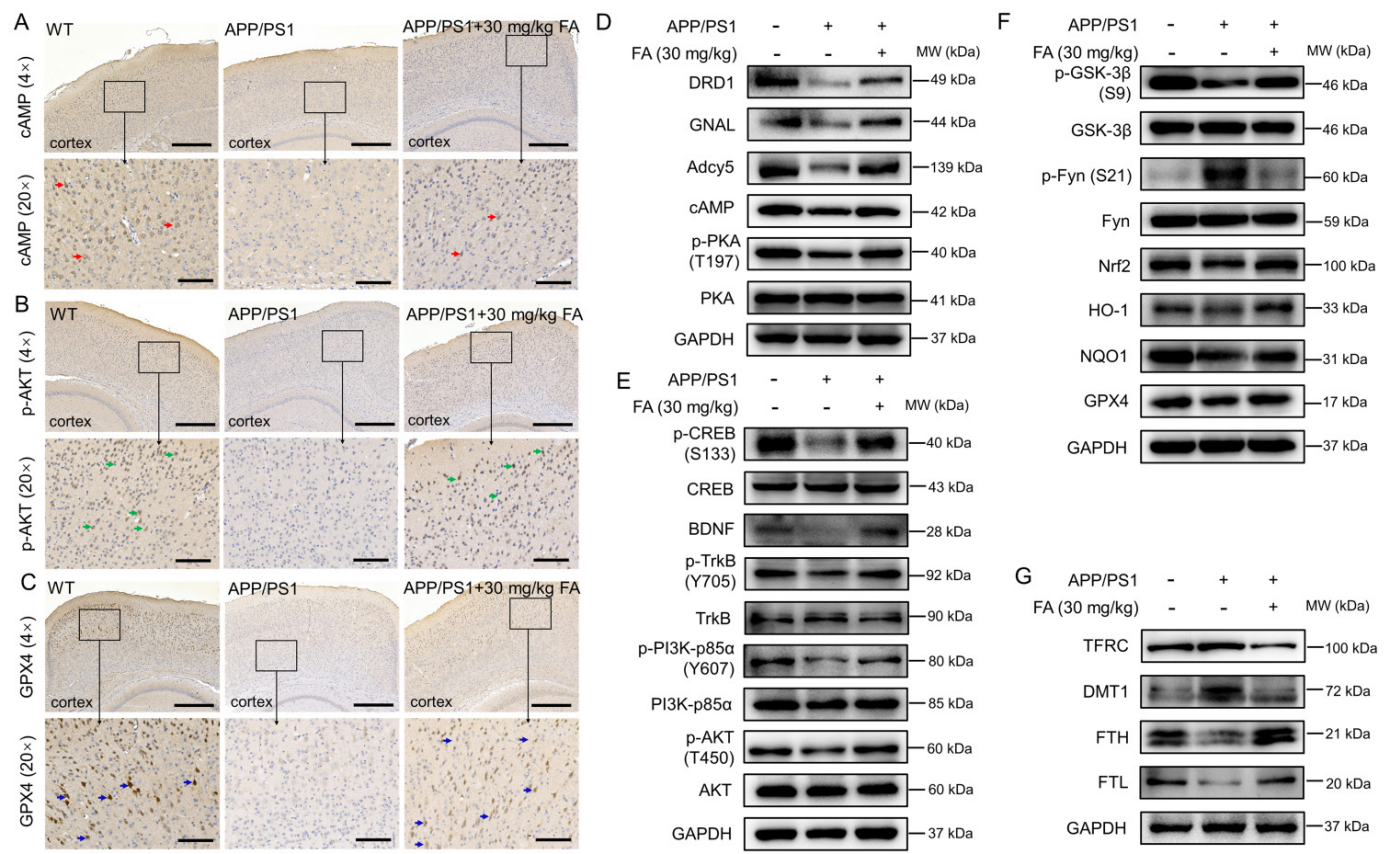
**FA treatment regulates the dopaminergic system and ferroptosis in APP/PS1 mice.** FA treatment increased the expression of (**A**) cAMP (red arrows), (**B**) p-AKT (green arrows), and (**C**) GPX4 (blue arrows) in the cortex in APP/PS1 mice (*n* = 3). Scale bar: 400 μm for 4× magnification and 100 μm for 20× magnification. (**D**) FA treatment enhanced the expression levels of DRD1, GNAL, Adcy5, cAMP, and p-PKA in the brains of APP/PS1 mice (*n* = 3). (**E**) FA treatment upregulated the phosphorylation of CREB, TrkB, PI3K, and AKT, and BDNF expression in the brains of APP/PS1 mice (*n* = 3). (**F**) FA treatment suppressed the expression of p-Fyn and upregulated the expression of p-GSK-3β, GPX4, Nrf2, and downstream proteins in the brains of APP/PS1 mice (*n* = 3). (**G**) FA treatment suppressed the expression of TFRC and DMT1 and upregulated the expression of FTH, and FTL in the brains of APP/PS1 mice (*n* = 3).

To further verify the proteomics results, immunohistochemistry was performed to detect the expression levels of cAMP, p-AKT, and GPX4 in the mouse brain. The expression of cAMP (*p* < 0.01) (Fig. 4A and Fig. S4A), p-AKT (*p* < 0.05) (Fig. 4B and Fig. S4B), and GPX4 (*p* < 0.01) (Fig. 4C and Fig. S4C) in the cerebral cortex was significantly increased in FA-treated APP/PS1 mice compared to that in vehicle-treated APP/PS1 mice.

Proteins related to the dopaminergic system and ferroptosis were analyzed. Compared with vehicle-treated APP/PS1 mice, FA-treated APP/PS1 mice exhibited higher levels of dopamine receptor D1 (DRD1) (a family of dopamine receptors that lead to protein kinase A (PKA) activation 28; *p* < 0.05), GNAL (encoding Gα(olf), which is related to increased cAMP levels 29; *p* < 0.01), Adcy5 (the main downstream effector of DA receptor signaling that is highly expressed in the striatum 24; *p* < 0.05), cAMP (a highly conserved second messenger that participates in DA-mediated learning 24; *p* < 0.001), and phospho (p)-PKA (a kinase positively coupled with dopamine D1 receptors that protects hippocampal neurons 28); *p* < 0.05) (Fig. 4D and Fig. S4D). These proteins are all related to the regulation of the dopaminergic system. Further, FA treatment increased the expression levels of p-cAMP-response element binding protein (CREB) (a protein that is essential for the formation of hippocampal-dependent long-term memory 30; *p* < 0.001), brain-derived neurotrophic factor (BDNF) (a downstream target gene of CREB that supports neuronal survival and promotes synaptic transmission 30; *p* < 0.01), p-tyrosine kinase receptor B (TrkB) (a downstream target gene of BDNF that protects neurons and synapses in its phosphorylated form 30; *p* < 0.001), p-phosphatidylinositol-3-kinase (PI3K) (an upstream regulator of AKT kinase 31; *p* < 0.05), and p-AKT (a proven target of ferroptosis regulation 31; *p* < 0.05) in the brains of APP/PS1 mice (Fig. 4E and Fig. S4E), implicating the regulation of key proteins in the dopaminergic terminal signaling pathway.

Nrf2 signaling can protect dopaminergic neurons against ferroptosis 32. Compared with vehicle-treated APP/PS1 mice, FA-treated APP/PS1 mice exhibited reduced phosphorylation of Fyn (its phosphorylated form accumulates in the nucleus and phosphorylates Nrf2 at tyrosine 568 33; *p* < 0.001) and enhanced expression levels of GPX4 (*p* < 0.001), p-glycogen synthase kinase-3β (GSK-3β) (an upstream regulator of Fyn kinase in the control of nuclear export of Nrf2 33; *p* < 0.01), Nrf2 (*p* < 0.05), and its downstream proteins (*p* < 0.01) (Fig. 4F and Fig. S4F), confirming the modulation of Nrf2 signaling.

FA treatment strongly suppressed the expression of transferrin receptor (TFRC) (a membrane receptor that mediates Fe^3+^ transport into cells 8; *p* < 0.001) and divalent metal-ion transporter-1 (DMT1) (a protein that transports Fe^2+^ into the cytoplasm 8; *p* < 0.05) and increased the expression of ferritin heavy chain (FTH) (a peptide responsible for capturing iron ions 34; *p* < 0.001), and ferritin light chain (FTL) (a peptide responsible for iron storage in cells 35; *p* < 0.001) in the brains of APP/PS1 mice (Fig. 4G and Fig. S4G), which further confirmed the alleviation of ferroptosis by FA treatment.

### FA treatment mitigated neuroinflammation in APP/PS1 mice

**Figure 5:**
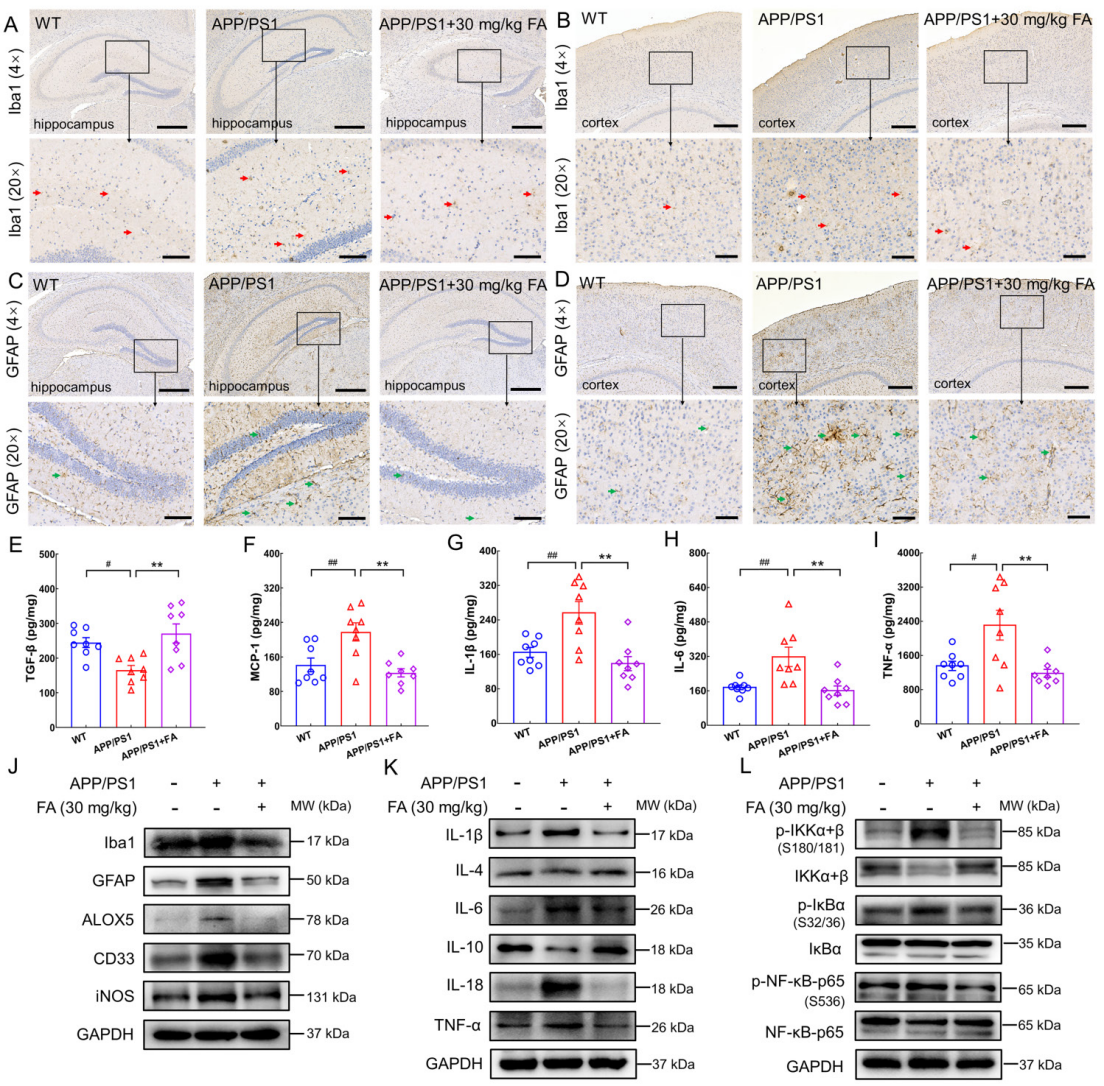
**FA treatment alleviates neuroinflammation in APP/PS1 mice.** FA treatment decreased the expression levels of Iba1 (red arrows) in the (**A**) hippocampus and (**B**) cerebral cortex in APP/PS1 mice (*n* = 3). FA treatment decreased the expression levels of GFAP (green arrows) in the (**C**) hippocampus and (**D**) cerebral cortex in APP/PS1 mice (*n* = 3). Scale bar: 400 µm for 4× magnification and 100 µm for 20× magnification. FA treatment upregulated the expression of (**E**) TGF-β and suppressed the expression levels of (**F**) MCP-1, (**G**) IL-1β, (**H**) IL-6, and (**I**) TNF-α in the brains of APP/PS1 mice (*n* = 8) detected with ELISA. (**J**) FA treatment downregulated the expression of Iba1, GFAP, ALOX5, CD33, and iNOS in the brains of APP/PS1 mice (*n* = 3). (**K**) FA treatment ameliorated the levels of ILs and TNF-α in the brains of APP/PS1 mice (*n* = 3). (**L**) FA treatment suppressed the phosphorylation of IKK, IκB, and NF-κB in the brains of APP/PS1 mice (*n* = 3). The data are presented as mean ± S.E.M. ^#^*p* < 0.05,^ ##^*p* < 0.01 vs. WT mice; **p < 0.01 vs. APP/PS1 mice.

In AD, neuroinflammation aggravates Aβ and tau pathology 11. The release of lipid metabolites in ferroptosis triggers inflammation, which reciprocally promotes ferroptosis by increasing iron deposition 36. FA treatment strongly suppressed the expression of ionized calcium-binding adapter molecule 1 (Iba1) (a microglial marker 6) and glial fibrillary acidic protein (GFAP) (an astrocytic marker 6) in the hippocampus (*p* < 0.001) (Fig. 5A, C and Fig. S5A, C) and cerebral cortex (*p* < 0.01) (Fig. 5B, D and Fig. S5B, D) in APP/PS1 mice. Double-labeling with GFAP and S100 calcium-binding protein B (S100B) further confirmed the inhibitory effect of FA treatment on the activation of astrocytes (Fig. S6).

An increase in pro-inflammatory cytokine levels and a decrease in anti-inflammatory cytokine levels exacerbate the degree of dementia in AD 37. Additionally, high expression of MCP-1 is observed in microglia surrounding senile plaques in the brains of patients with AD 38. ELISA results revealed a marked increase in TGF-β expression (*p* < 0.01) (Fig. 5E) and decreased MCP-1 (*p* < 0.01) (Fig. 5F), IL-1β (*p* < 0.01) (Fig. 5G), IL-6 (*p* < 0.01) (Fig. 5H), and TNF-α (*p* < 0.01) (Fig. 5I) expression levels in the brains of FA-treated APP/PS1 mice relative to those of vehicle-treated APP/PS1 mice.

Compared to WT mice, APP/PS1 mice exhibited higher levels of Iba1 (*p* < 0.05), GFAP (*p* < 0.01), arachidonate 5-lipoxygenase (ALOX5) (an enzyme that participates in AD-related neuroinflammation by enhancing Iba1 and GFAP 39; *p* < 0.001), myeloid cell surface antigen CD33 (CD33) (a transmembrane sialic acid-binding receptor located on the surface of microglia that promotes Aβ pathology 40; *p* < 0.05), and inducible nitric oxide synthase (iNOS) (an enzyme that produces NO which can be toxic to neurons and is upregulated in the brains of patients with AD 37; *p* < 0.001) (Fig. 5J and Fig. S5E). FA treatment markedly decreased the expression levels of these proteins (*p* < 0.05) (Fig. 5J and Fig. S5E).

Furthermore, FA treatment induced an upregulation of anti-inflammatory factors, including IL-4 (*p* < 0.01) and IL-10 (*p* < 0.001) and a concomitant downregulation of proinflammatory factors, including IL-1β (*p* < 0.01), IL-6 (*p* < 0.01), IL-18 (*p* < 0.01), and TNF-α (*p* < 0.01), as well as p-IκB kinase (IKK) (*p* < 0.05), p-IκB (*p* < 0.01), and p-NF-κB (*p* < 0.01) (Fig. 5K, L and Fig. S5F, G). Collectively, these data suggested that FA treatment suppressed neuroinflammation in the brains of APP/PS1 mice.

### FA treatment attenuated ferroptosis and neuroinflammation in erastin-exposed HT22 cells

**Figure 6:**
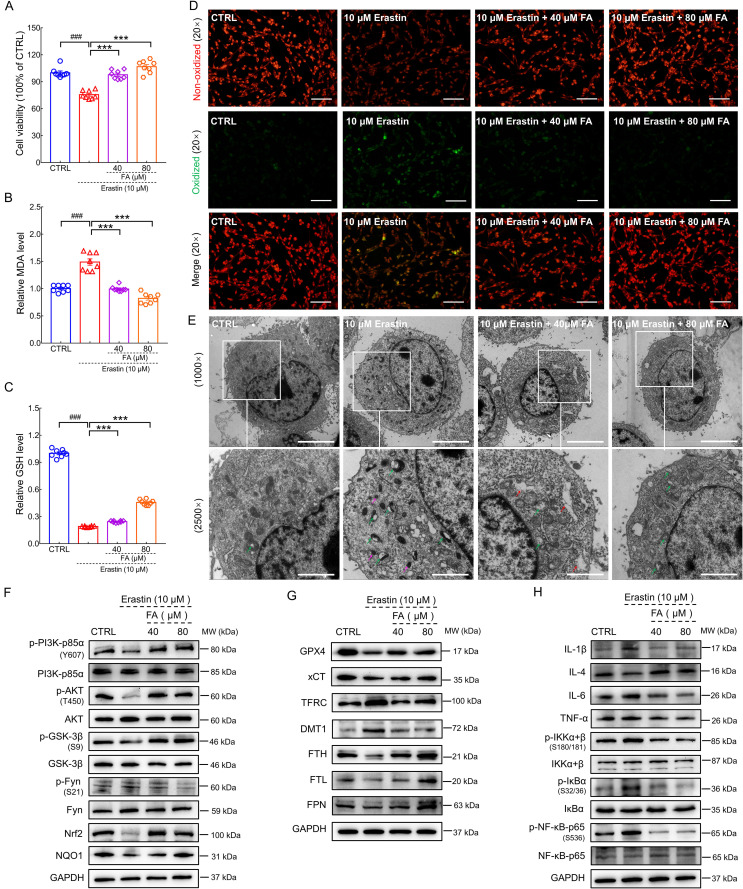
** FA alleviates ferroptosis-related inflammation in erastin-exposed HT22 cells.** FA treatment (**A**) improved cell viability, (**B**) decreased MDA levels, and (**C**) increased GSH levels in erastin-exposed HT22 cells (*n* = 8). (**D**) FA treatment suppressed lipid peroxidation in erastin-exposed HT22 cells (*n* = 3) as determined using BODIPY 581/591 C11. Red and green fluorescence signals represent non-oxidized and oxidized states, respectively. Scale bar: 100 µm. (**E**) Ultrastructure of HT22 cells in each group was analyzed using TEM (*n* = 3). Green, pink, and red arrows indicate disrupted mitochondrial cristae, increased mitochondrial electron density, and rough endoplasmic reticulum expansion. Scale bar: 5 µm for 1000× magnification and 2 µm for 2500× magnification. (**F**) FA treatment upregulated the expression of p-PI3K, p-AKT, p-GSK-3β, Nrf2, and NQO1, and downregulated the expression of p-Fyn in erastin-exposed HT22 cells (*n* = 3). (**G**) FA treatment upregulated the expression of GPX4, xCT, FTH, FTL, and FPN, and suppressed the expression of TFRC and DMT1 in erastin-exposed HT22 cells (*n* = 3). (**H**) FA treatment downregulated the expression of IL-1β, IL-6, and TNF-α; suppressed the phosphorylation of IKK, IκB, and NF-κB; and upregulated the expression of IL-4 in erastin-exposed HT22 cells (*n* = 3). The data are presented as mean ± S.E.M. ^###^*p* < 0.001 vs. CTRL HT22 cells; ***p < 0.001 vs. erastin-exposed HT22 cells.

As a classic ferroptosis inducer, erastin changes the permeability of the outer mitochondrial membrane by binding voltage-dependent anion channel 2/3 to reduce the rate of nicotinamide adenine dinucleotide oxidation, thereby inducing ferroptosis 41. Further, the inhibition of GPX4 increases lipid ROS production, eventually leading to cell ferroptosis 42. FA pretreatment significantly improved cell viability in erastin-exposed HT22 cells (*p* < 0.001) (Fig. 6A). NAD(P)H-GSH-GPX4 signaling is a classic pathway that inhibits ferroptosis. GSH acts as an electron donor to reduce toxic phospholipid hydroperoxide to non-toxic phospholipid alcohol. This maintains GPX4 activity and reduces lipid peroxidation which can be broken down into MDA, an end product that is severely toxic to cells 8. FA treatment significantly suppressed MDA levels (*p* < 0.001) (Fig. 6B) and increased GSH levels (*p* < 0.001) (Fig. 6C) in erastin-stimulated HT22 cells. BODIPY 581/591 C11 staining revealed that FA treatment inhibited erastin-induced lipid ROS production in HT22 cells (*p* < 0.01) (Fig. 6D and Fig. S7).

Ferroptosis results in morphological changes in mitochondrial ultrastructure 43. TEM analysis revealed that mitochondria in erastin-stimulated cells had smaller volume, higher electron density, and disrupted mitochondrial cristae; these pathological alterations in morphology were ameliorated by FA treatment (Fig. 6E). In erastin-exposed HT22 cells, FA treatment significantly attenuated p-Fyn expression (*p* < 0.05) and upregulated p-PI3K (*p* < 0.05), p-AKT (*p* < 0.01), p-GSK-3β (*p* < 0.05), Nrf2 (*p* < 0.05), and NAD(P)H quinone dehydrogenase 1 (NQO1) (*p* < 0.05) expression (Fig. 6F and Fig. S8A). Similar to the *in vivo* data, FA treatment upregulated the expression of GPX4 (*p* < 0.05), cystine/glutamate transporter (xCT) (*p* < 0.01), FTH (*p* < 0.05), FTL (*p* < 0.05), and ferroportin (FPN) (the only known protein in mammals that underscores export of iron 8; *p* < 0.05) and suppressed the expression of TFRC (*p* < 0.05) and DMT1 (*p* < 0.05) (Fig. 6G and Fig. S8B). FA treatment suppressed the activation of NF-κB signaling (*p* < 0.05), which further regulated the expression of ILs (*p* < 0.05) and TNF-α (*p* < 0.05) (Fig. 6H and Fig. S8C). In conjunction with the *in vivo* results, these findings indicated that FA treatment exerted anti-ferroptosis and anti-neuroinflammatory effects.

### Anti-ferroptosis and anti-neuroinflammatory effects of FA treatment were mediated by the Nrf2/GPX4 axis

**Figure 7:**
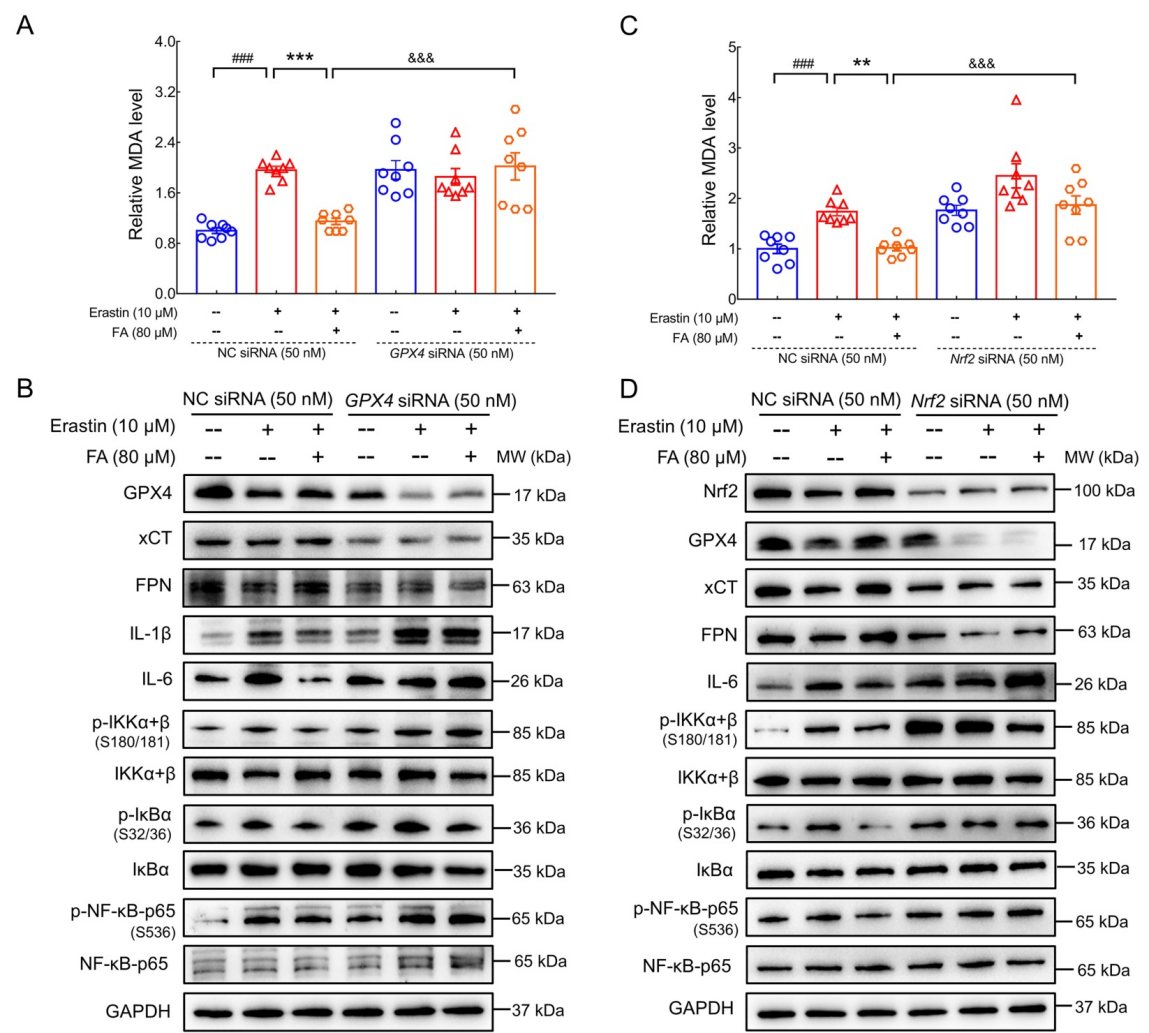
** GPX4 and Nrf2 are involved in the anti-ferroptosis and anti-neuroinflammatory effects of FA treatment.** (**A**) FA treatment-induced decreases in MDA levels were blocked in *GPX4* siRNA-transfected erastin-exposed HT22 cells (*n* = 8). (**B**) FA treatment-induced increases in GPX4, xCT and FPN levels; reduction in IL-6 and IL-1β levels; and suppression of NF-κB signaling were abolished in *GPX4* siRNA-transfected erastin-exposed HT22 cells (*n* = 3). (**C**) FA treatment-induced decreases in MDA levels were blocked in *Nrf2* siRNA-transfected erastin-exposed HT22 cells (*n* = 8). (**D**) FA treatment-induced upregulation of Nrf2, GPX4, xCT, and FPN was abolished and NF-κB signaling was activated in *Nrf2* siRNA-transfected erastin-exposed HT22 cells (*n* = 3). The data are presented as mean ± S.E.M. ^###^*p* < 0.001 vs. CTRL HT22 cells transfected with NC siRNA; **p < 0.01, ***p < 0.001 vs. erastin-exposed HT22 cells transfected with NC siRNA; ^&&&^*p* < 0.001 vs. FA-treated HT22 cells transfected with NC siRNA.

Increased iron levels in the brain induce pro-inflammatory factor production by microglia; conversely, iron accumulation is an established consequence of inflammation 7. Nevertheless, the specific regulatory processes linking neuroinflammation and ferroptosis have yet to be elucidated. A recent study reported that targeting the Nrf2/GPX4 axis effectively regulated ferroptosis 44. GPX4 plays a key role in preventing ferroptosis by reducing phospholipid hydroperoxide, thus inhibiting lipid peroxidation. Accordingly, GPX4 activation inhibits NF-κB signaling in lipid peroxidation-mediated diseases 13. The regulatory effects of FA treatment on MDA levels (*p* < 0.001) (Fig. 7A); expression of GPX4 (*p* < 0.001), xCT (*p* < 0.05), FPN (*p* < 0.01), and ILs (*p* < 0.01); and IKK/IκB/NF-κB activation (*p* < 0.05) were all strongly abolished in *GPX4* siRNA-transfected erastin-exposed HT22 cells (Fig. 7B and Fig. S9). Furthermore, the molecular docking results showed that FA possessed an affinity for GPX4 with a binding energy of -6.35 kcal/mol, FA formed hydrogen bonds with GPX4 via GLN-45, CYS-46, GLY-47, LYS-48, ARG-80, and GLN-81. The interaction between FA and GPX4 formed a binding pocket (Fig. S10).

Nrf2 is a negative regulator of Aβ pathology 45 and modulates ferroptosis by regulating endogenous anti-ferroptosis components, including upregulation of xCT, activation of FTH, and heme oxygenase-1 (HO-1), and restoration of GPX4 activity 12. The regulatory effects of FA treatment on MDA levels (*p* < 0.001) (Fig. 7C); expression of Nrf2 (*p* < 0.001), GPX4 (*p* < 0.001), xCT (*p* < 0.01), FPN (*p* < 0.001), and IL-6 (*p* < 0.01); and IKK/IκB/NF-κB activation (*p* < 0.01) were all strongly abolished in *Nrf2* siRNA-transfected erastin-exposed HT22 cells (Fig. 7D and Fig. S11). These data are consistent with previous observations that Nrf2 regulates ferroptosis and neuroinflammation by influencing GPX4 expression. Collectively, these data confirmed the involvement of the Nrf2/GPX4 axis in FA-mediated anti-ferroptosis and anti-neuroinflammation and provided insight into the crosstalk between ferroptosis and neuroinflammation.

## Discussion

In this study, we first expounded the protective properties of FA in AD based on its effects in Aβ-exposed N2a cells, erastin-stimulated HT22 cells, and LPS-stimulated BV2 cells as well as its beneficial effects on memory and cognitive abilities in APP/PS1 mice with AD-like behaviors. Label-free quantitative proteomics analysis demonstrated that FA treatment modulates the expression of Adcy5 and GNAL, which affect learning by altering cAMP-related dopamine signaling 24, 25, subsequently suppressing neuroinflammation and preventing ferroptosis by inhibiting NF-κB activity and reducing iron accumulation 26, 27. Furthermore, the Nrf2/GPX4 axis was confirmed to play key roles in FA-mediated anti-neuroinflammation and anti-ferroptosis.

To date, the pathogenesis of AD has not been fully elucidated, and the development of effective treatments has been challenging 3. Natural products, such as marine-derived oligosaccharides (GV-971), have been identified as potential therapeutic agents 46. Extracellular senile plaques composed of Aβ peptides have been reported as a key marker of the initiation of the pathogenic cascade of AD 47, and an imbalance between production and clearance may induce synaptic dysfunction and neuronal damage 48. The monoclonal antibody aducanumab targets soluble and insoluble neurotoxic Aβ oligomers and was approved by the US FDA for the treatment of AD in June 2021 5. Activated microglia associated with Aβ neurotoxicity cause chronic inflammation and aggravate neuronal damage 49. GV-971 has been reported to inhibit intestinal dysbiosis and regulate the accumulation of phenylalanine/isoleucine and suppress neuroinflammation, thereby improving cognitive impairment, which confirms the key role of neuroinflammation in the pathological process underscoring AD 46. FA treatment reduced Aβ deposition and exhibited anti-neuroinflammatory effects, as demonstrated by the suppression of pro-inflammatory cytokines and enhancement of anti-inflammatory cytokines in several cell lines and APP/PS1 mice.

Significant reductions in DA and DA metabolite levels have been observed in several regions of the post-mortem AD brain 50. These changes are associated with behavioral symptoms such as apathy and depression and may contribute to cognitive decline 51. In the Tg2576 mouse model of AD, age-dependent alterations in dopaminergic neuron loss in the ventral tegmental area occur prior to the formation of amyloid plaques, leading to memory deficits 52. Rotigotine, a DA agonist, has demonstrated beneficial effects on frontal-executive function in a phase 2 trial for AD 4. The selective DRD1 agonist SKF81297 is thought to be involved in ameliorating hippocampal synaptic damage and improving spatial memory in AD mouse models 53. DRD1 is the most abundant DA receptor in the central nervous system, and its downstream signaling involves the binding of Gαolf, encoded by the *GNAL* gene, to guanosine triphosphate. This results in Adcy5 activation and subsequent cAMP production 25. Allosteric activation of cAMP promotes cAMP-dependent PKA phosphorylation of CREB 54, thereby initiating the transcription and translation of CREB target genes, which are essential for the formation of long-term memories 55. Impaired cAMP signaling (including p-CREB) has been observed in the brains of AD patients and relevant mouse models 30, 56. The enhancement of phosphorylated CREB ameliorates memory impairments in AD mouse models 57, which is consistent with the results of our study. Moreover, CREB mediates the formation of short-term memories by upregulating BDNF 58. Accordingly, the combination of BDNF and its endogenous receptor, TrkB, may cause autophosphorylation of the intracellular domain of tyrosine residues, which initiates PI3K/AKT activation and promotes neuronal survival 59. The present data confirm the involvement of dopaminergic signaling in FA-mediated anti-AD effects.

BDNF has been proposed to activate Nrf2 in astrocytes via the truncated TrkB.T1 and p75NTR receptor complex in astrocytes to protect dopaminergic neurons from ferroptosis 32. The non-oxidized form of DA serves as a strong inhibitor of ferroptosis by inhibiting intracellular iron accumulation and iron-mediated ROS production or by reducing GSH consumption and GPX4 degradation 26. Intracellular iron is stored in ferritin, which is composed of FTH and FTL, or exported by FPN. Imbalances in iron input, storage, and output may affect susceptibility to lipid peroxidation 8, 60. During this process, TFRC and DMT1 regulate the translocation of iron into cells 61. Due to the enrichment of polyunsaturated fatty acid in the brains of AD patients 60, lipid ROS alter the structure of the lipid bilayer and destroys its barrier function. The formation of lipid peroxides further amplifies ROS signals and decomposes these products to produce the toxic derivative, MDA. MDA reacts with DNA bases or proteins, causing severe cytotoxicity. GPX4 inhibits lipoxygenase-mediated lipid peroxidation by reducing phospholipid hydrogen peroxide and plays a key role in preventing ferroptosis 8. In this study, FA treatment significantly reduced intracellular iron content by partially reducing iron entry and promoting iron output. These effects were associated with a reduction in lipid peroxidation in cell lines and APP/PS1 mice.

In pathological conditions of neurodegenerative diseases, iron overload triggers microglial polarization to the pro-inflammatory (M1) phenotype via ROS, which increases the secretion of TNF-α and IL-1β and promotes neuroinflammation 62. IL-1β and TNF-α upregulate TFRC and DMT1 and downregulate FPN, resulting in increased ferrous iron influx and reduced iron efflux in cells 63. Consequently, neuroinflammation and iron interact to form an amplified ROS production circuit, leading to neuronal ferroptosis 62. Metabolic dysfunction of microglia and astrocytes leads to Aβ accumulation, which in turn activates microglia and astrocytes, and activated glial cells release neuroinflammatory mediators, including IL-1β and IL-6 via Toll-like receptors and NF-κB signaling pathways 64, 65. IL-1β induces IL-6 production by astrocytes and neurons, which in turn modulates behavior and cognitive function in rats administered with IL-6 into the bilateral hippocampus and in IL-6 knockout mice 66. Our results demonstrated that FA treatment exerted anti-neuroinflammatory properties by targeting microglia and astrocytes via IKK/IκB/NF-κB signaling.

Based on previous research demonstrating the involvement of anti-ferroptosis and anti-neuroinflammation in AD treatment, we investigated FA-mediated anti-AD effects. GPX4 stimulates the release of anti-inflammatory lipid mediators in the inflammation-related arachidonic acid metabolic network and inhibits the NF-κB pathway activated by TNF or IL-1 67. An increase in GFAP expression levels was observed in adult mice that were deficient in GPX4 10, which is consistent with our observation that GPX4 is a promising target for suppressing inflammation. Nrf2 has been reported to regulate neuroinflammation and ferroptosis and influence GPX4 expression 9. siRNA-induced silencing of Nrf2 expression blocked melatonin-induced upregulation of GPX4 and xCT expression in MC3T3 cells 68. Moreover, the concentration of supernatant TNF-α was increased and that of supernatant IL-4 and IL-10 was decreased in Nrf2-silenced BV2 cells 69. These findings are consistent with the observation that *Nrf2* siRNA abrogated FA-mediated upregulation of GPX4, xCT, and FPN and NF-κB pathway inactivation. Collectively, these results indicate that FA treatment protects against erastin-induced ferroptosis by activating the Nrf2/GPX4 axis, and its suppression of ferroptosis exerts positive regulatory effects on neuroinflammation.

This study has several limitations. We only measured ferroptosis and neuroinflammation-related proteins and factors in the brains of APP/PS1 mice at a single time point rather than at different time stages. As such, we did not assess whether ferroptosis occurs prior to or concurrently with neuroinflammation, and it remains unclear whether a feedback loop between these processes exists. Additionally, the relationship between Nrf2/GPX4 and dopaminergic terminal signaling pathways, as well as IKK/IκB/NF-κB signaling, remains to be explored.

## Conclusions

This study demonstrated that FA treatment exerted anti-AD properties via modulation of ferroptosis-mediated neuroinflammation by targeting the activation of the Nrf2/GPX4 axis. Our data suggest that inactivation of the Nrf2/GPX4 axis activates NF-κB signaling, which further aggravates neuroinflammation (Fig. S12). Our study systematically demonstrates the multitarget protective effects of FA and highlights its therapeutic potential as a promising drug component for the treatment of AD.

## Supplementary Material

Supplementary methods, figures and tables.

## Abbreviations

AD: Alzheimer's disease
Aβ: amyloid-β
Adcy5: adenylate cyclase 5
AKT: protein kinase B
ALOX5: arachidonate 5-lipoxygenase
BDNF: brain-derived neurotrophic factor
cAMP: cyclic adenosine monophosphate
CREB: cAMP-response element binding protein
DRD1: dopamine receptor D1
DA: dopamine
DMT1: divalent metal-ion transporter-1
ELISA: enzyme-linked immunosorbent assay
FA: forsythoside A
FTH: ferritin heavy chain
FTL: ferritin light chain
FPN: ferroportin
GPX4: glutathione peroxidase 4
GNAL: guanine nucleotide-binding protein G(olf) subunit α
GFAP: glial fibrillary acidic protein
H&E: hematoylin and eosin
HO-1: heme oxygenase-1
IκB: inhibitor of NF-κB
IKK: IκB kinase
Iba1: ionized calcium binding adapter molecule 1
iNOS: inducible nitric oxide synthase
IL: interleukin
LPS: lipopolysaccharide
MWM: Morris water maze
MMP: mitochondrial membrane potential
MDA: malondialdehyde
MCP-1: monocyte chemoattractant protein-1
NO: nitric oxide
NF-κB: nuclear factor-κB
Nrf2: nuclear factor erythroid 2-related factor 2
NQO1: NAD(P)H quinone dehydrogenase 1
PBS: phosphate buffer solution
PKA: protein kinase A
GSK-3β: glycogen synthase kinase-3β
PI3K: phosphatidylinositol-3-kinase
ROS: reactive oxygen species
RNAi: RNA interference
RIPA: radio immunoprecipitation assay
siRNA: small interfering RNA
TNF: tumor necrosis factor
TEM: transmission electron microscopy
TGF-β: transforming growth factor-β
TFRC: transferrin receptor
TrkB: tyrosine kinase receptor B
xCT: cystine/glutamate transporter
