# Supplementary material for: Forsythoside A Mitigates Alzheimer's-like Pathology by Inhibiting Ferroptosis-mediated Neuroinflammation via Nrf2/GPX4 Axis Activation
Source: Int J Biol Sci. 2022 Feb 28;18(5):2075–90. doi: 10.7150/ijbs.69714 (PMC8935224; doi:10.7150/ijbs.69714)
Supplement: Supplementary file 1 — Supplementary methods, figures and tables. [file ijbsv18p2075s1.pdf]

Supplemental Data

Methods

1. Hematoxylin and Eosin (H&E) staining

Pathological changes in the liver, spleen, and kidney tissues were analyzed using H&E staining as described in our previous study [1]. Tissues were fixed in 4% paraformaldehyde and serially dehydrated in ethanol. After embedding in paraffin, 0.5-μm-thick sections were collected and stained with H&E. The sections were observed using an optical microscope (BX51, Olympus, Tokyo, Japan).

2. Immunofluorescence

The paraformaldehyde-fixed brain slides were incubated with GFAP and S100B (Table S1) followed by the combination of fluorescein-labelled secondary antibodies (Table S1). Then images were acquired through a fluorescence microscope (BX53, Olympus, Tokyo, Japan).

3. Molecular docking

AutoDock 4.2 was used for molecular docking, which predicts the binding pattern and affinity of small molecular ligands by studying their interactions with protein receptors. Docking FA into GPX4 based on the determined substrate binding site of GPX4 crystal structure, and molecular docking calculation using the Lamarckian genetic algorithm, the structure with the lowest energy is selected as the analysis object from the one with the most clustering results.

Table S1. Details of antibodies used in immunohistochemistry, western blot and immunofluorescence

Antibody	Molecular weight	Catalog number	Dilution	Application	Company	Area
Aβ	4.4 kDa	bs-0107R	1:800	Immunohistochemistry	Bioss	Beijing, China
p-Tau (S396)	79 kDa	ab109390	1:4000	Immunohistochemistry	Abcam	Cambridge, MA, USA
cAMP	42 kDa	ab76238	1:150	Immunohistochemistry	Abcam	Cambridge, MA, USA
p-AKT (T450)	60 kDa	BM4721	1:100	Immunohistochemistry	Boster	Wuhan, China
GPX4	17 kDa	BA3802-1	1:100	Immunohistochemistry	Boster	Wuhan, China
Iba1	17 kDa	10904-1-AP	1:400	Immunohistochemistry	Proteintech	Wuhan, China
GFAP	50 kDa	PB9082	1:400	Immunohistochemistry	Boster	Wuhan, China
DRD1	49 kDa	A2893	1:1000	Western blot	ABclonal	Wuhan, China
GNAL	44 kDa	abs141187	1:750	Western blot	Absin	Shanghai, China

Adcy5	139 kDa	bs-3922R	1:1000	Western blot	Bioss	Beijing, China
cAMP	42 kDa	ab76238	1:2000	Western blot	Abcam	Cambridge, MA, USA
p-PKA (T197)	40 kDa	ab75991	1:3000	Western blot	Abcam	Cambridge, MA, USA
PKA	41 kDa	06-903	1:1000	Western blot	Millipore	Darmstadt, Germany
p-CREB (S133)	40 kDa	ab32096	1:5000	Western blot	Abcam	Cambridge, MA, USA
CREB	43 kDa	A11064	1:750	Western blot	ABclonal	Wuhan, China
BDNF	28 kDa	abs115532	1:1000	Western blot	Absin	Shanghai, China
p-TrkB (Y705)	92 kDa	ab229908	1:1000	Western blot	Abcam	Cambridge, MA, USA
TrkB	90 kDa	A2099	1:1000	Western blot	ABclonal	Wuhan, China
p-PI3K-p85α (Y607)	80 kDa	AF3241	1:1000	Western blot	Affinity	Cincinnati, OH, USA
PI3K-p85α	85 kDa	A4992	1:1000	Western blot	ABclonal	Wuhan, China
p-AKT (T450)	60 kDa	ab108266	1:5000	Western blot	Abcam	Cambridge, MA, USA

AKT	60 kDa	A18120	1:750	Western blot	ABclonal	Wuhan, China
TFRC	100 kDa	A5865	1:1000	Western blot	ABclonal	Wuhan, China
DMT1	72 kDa	A10231	1:1500	Western blot	ABclonal	Wuhan, China
FTH	21 kDa	A19544	1:1000	Western blot	ABclonal	Wuhan, China
FTL	20 kDa	A1768	1:1000	Western blot	ABclonal	Wuhan, China
p-GSK-3β (S9)	46 kDa	AP1088	1:1000	Western blot	ABclonal	Wuhan, China
GSK-3β	46 kDa	A2081	1:1000	Western blot	ABclonal	Wuhan, China
p-Fyn (S21)	60 kDa	AP0510	1:1000	Western blot	ABclonal	Wuhan, China
Fyn	59 kDa	A9165	1:1000	Western blot	ABclonal	Wuhan, China
Nrf2	100 kDa	A1244	1:1000	Western blot	ABclonal	Wuhan, China
HO-1	33 kDa	A19062	1:1000	Western blot	ABclonal	Wuhan, China
NQO1	31 kDa	A19586	1:1000	Western blot	ABclonal	Wuhan, China

GPX4	17 kDa	A11243	1:1000	Western blot	ABclonal	Wuhan, China
Iba1	17 kDa	A19776	1:1000	Western blot	ABclonal	Wuhan, China
GFAP	50 kDa	80788S	1:1000	Western blot	Cell Signaling Technology	Beverly, MA, USA
ALOX5	78 kDa	A2877	1:1000	Western blot	ABclonal	Wuhan, China
CD33	70 kDa	A2059	1:1000	Western blot	ABclonal	Wuhan, China
iNOS	131 kDa	A3200	1:750	Western blot	ABclonal	Wuhan, China
IL-1β	17 kDa	A1112	1:1000	Western blot	ABclonal	Wuhan, China
IL-4	16 kDa	A4988	1:1000	Western blot	ABclonal	Wuhan, China
IL-6	26 kDa	A0286	1:1000	Western blot	ABclonal	Wuhan, China
IL-10	18 kDa	A2171	1:1000	Western blot	ABclonal	Wuhan, China
IL-18	18 kDa	A1115	1:1000	Western blot	ABclonal	Wuhan, China

TNF-α	26 kDa	17590-1-AP	1:1000	Western blot	Proteintech	Wuhan, China
p-IKKα+β (S180/181)	85 kDa	AF3013	1:1000	Western blot	Affinity	Cincinnati, OH, USA
IKKα+β	75/87 kDa	ab178870	1:1000	Western blot	Abcam	Cambridge, MA, USA
IKKα+β	85 kDa	A2062	1:1000	Western blot	ABclonal	Wuhan, China
p-IκBα (S32/36)	36 kDa	ab12135	1:500	Western blot	Abcam	Cambridge, MA, USA
p-IκBα (S32/36)	36 kDa	AF2002	1:1000	Western blot	Affinity	Cincinnati, OH, USA
IκBα	35 kDa	ab32518	1:4000	Western blot	Abcam	Cambridge, MA, USA
p-NF-κB-p65 (S536)	65 kDa	ab76302	1:4000	Western blot	Abcam	Cambridge, MA, USA
NF-κB-p65	65 kDa	ab16502	1:4000	Western blot	Abcam	Cambridge, MA, USA
xCT	35 kDa	A13685	1:1000	Western blot	ABclonal	Wuhan, China
FPN	63 kDa	A14884	1:1000	Western blot	ABclonal	Wuhan, China
GAPDH	37 kDa	E-AB-20032	1:4000	Western blot	Elabscience	Wuhan, China

goat anti-mouse		E-AB-1001	1:4000	Western blot	Elabscience	Wuhan, China
goat anti- rabbit		E-AB-1003	1:4000	Western blot	Elabscience	Wuhan, China
goat anti-rabbit		BA1003	1:150	Immunohistochemistry	Boster	Wuhan, China
GFAP	49kDa	GB12096	1:500	Immunofluorescence	Servicebio	Wuhan, China
S100B	11kDa	GB11359	1:500	Immunofluorescence	Servicebio	Wuhan, China
Cy3 conjugated goat anti-mouse IgG (H+L)		GB21301	1:300	Immunofluorescence	Servicebio	Wuhan, China
Alexa Fluor® 488-conjugated goat anti-rabbit IgG (H+L)		GB25303	1:400	Immunofluorescence	Servicebio	Wuhan, China

Table S2. Proteins with significantly different expression levels in proteomics

Number	Protein. names	Unique. peptides	fc. WT - APP/PS1	fc. APP/PS1+FA -APP/PS1
**Upregulated proteins by FA (Number: 10)**				
1	Clasp1	8	108.8679433	116.7574448
2	Cox7c	2	75.9464048	74.10680885
3	Hnrnpdl	5	27.7350564	20.27176613
4	GNAL	4	7.821399026	3.006802096
5	Adcy5	13	7.603245852	4.379024494
6	Pde10a	11	5.52792181	3.813609875
7	Gnb4	5	4.879425182	5.864464034
8	Pfdn6	2	4.31827841	4.952922853
9	Mpp3	6	2.011522507	1.6493573
10	Chmp1b	2	1.604717166	1.546388726
**Downregulated proteins by FA (Number: 5)**				
1	Rbp1	3	0.63146	0.440041
2	Tbcd	4	0.631048	0.517827
3	Khdrbs3	3	0.27188	0.420554
4	Cacna1a	5	0.080042	0.09214
5	Tmsb4x	4	0.006312	0.004574

fc. WT-APP/PS1: the ratio of protein between WT mice and vehicle-treated APP/PS1 mice;

fc. APP/PS1+FA-APP/PS1: the ratio of protein between FA-treated APP/PS1 mice and vehicle-treated APP/PS1 mice.

Figure List

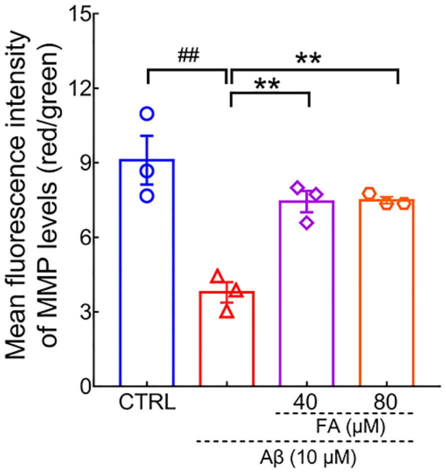

Figure S1. The quantification of the mean fluorescence intensity of MMP levels (n = 3). The data are shown as means ± S.E.M. $^{\#\#}p < 0.01$ vs. CTRL N2a cells, $^{**}p < 0.01$ vs. $A\beta_{1\text{-}42}$-exposed N2a cells.

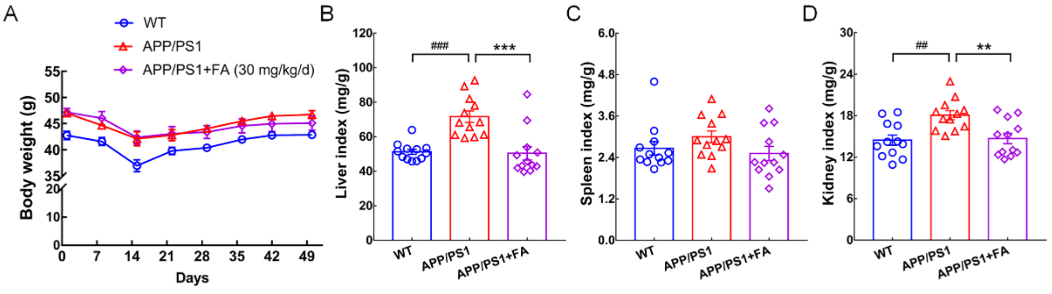

Figure S2. Observation of mice body weight and organs index. (**A**) The changes on body weight of APP/PS1 mice during the experimental period (n = 12). (**B**) FA reversed the increase in liver index of APP/PS1 mice (n = 12). (**C**) FA showed no significant effect on the spleen index of mice (n = 12). (**D**) FA downregulated the kidney index of APP/PS1 mice (n = 12). The data are shown as means ± S.E.M. $^{\#\#}p < 0.01$, $^{\#\#\#}p < 0.001$ vs. WT mice, $^{**}p < 0.01$, $^{***}p < 0.001$ vs. APP/PS1 mice.

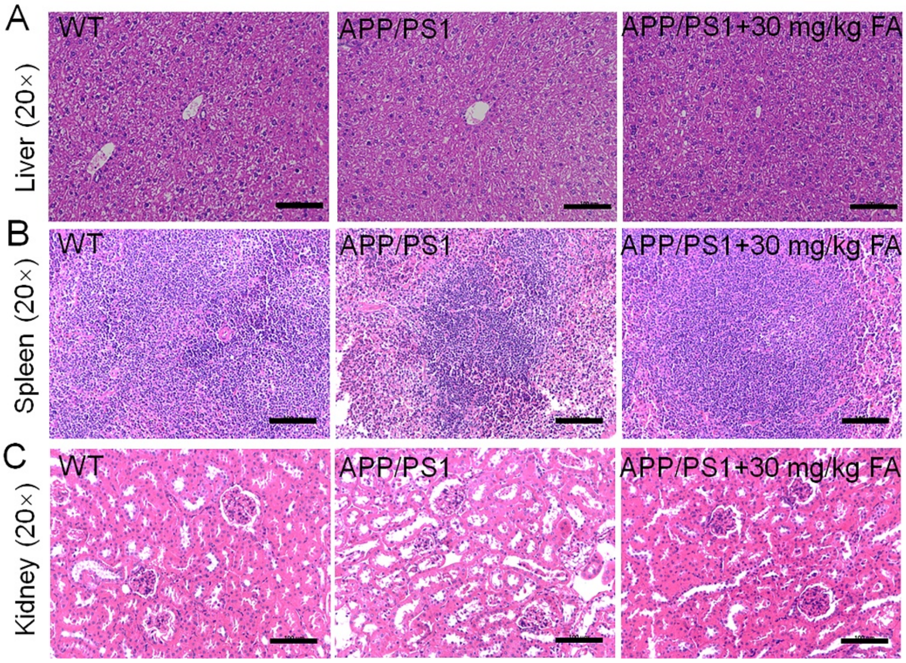

Figure S3. H&E staining of liver, spleen, and kidney (scale bar: 100 μm) (n = 3).

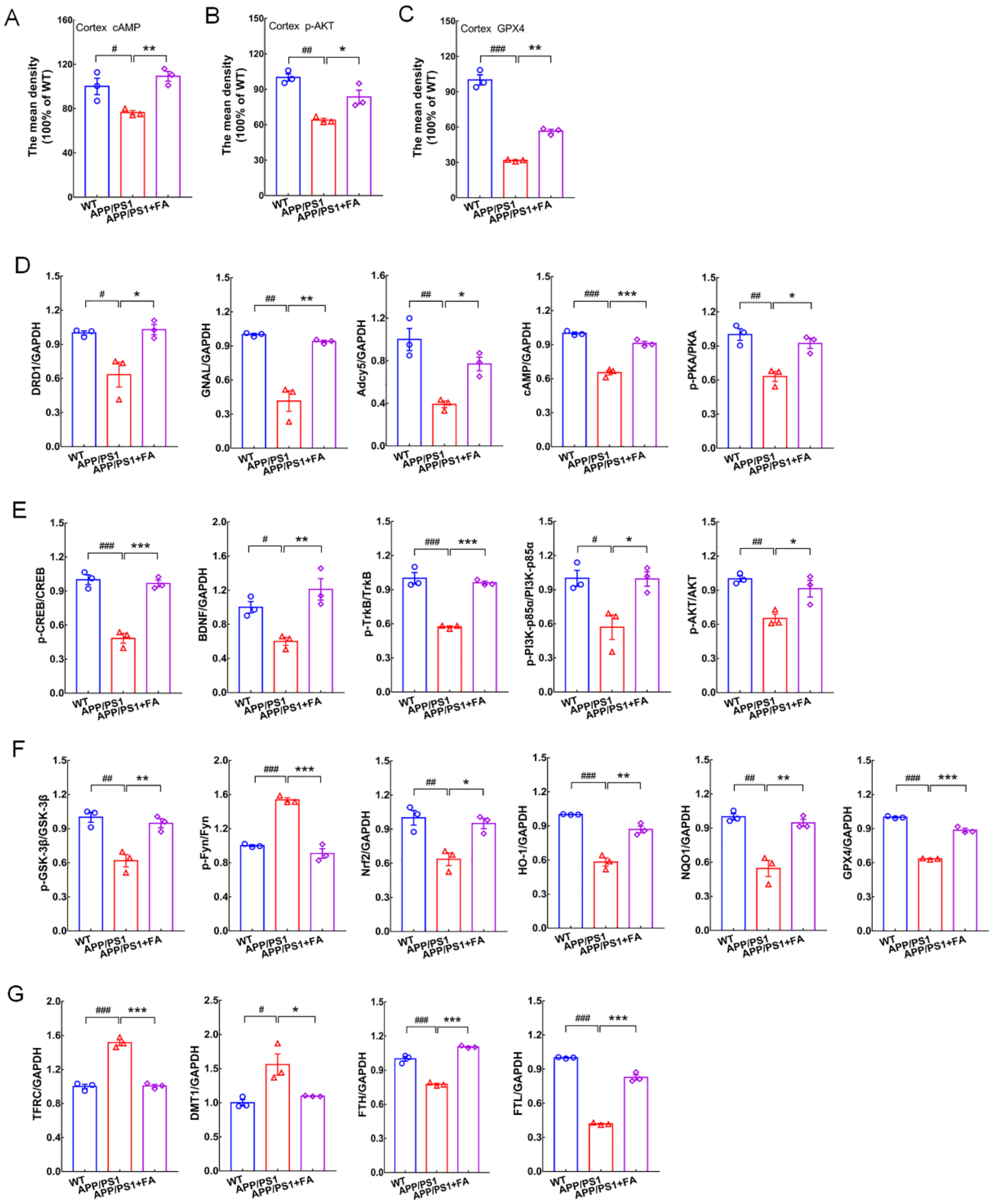

Figure S4. FA regulated the dopaminergic system and ferroptosis in brains of APP/PS1 mice. (**A** to **C**) Quantification of the mean density of (**A**) cAMP, (**B**) p-AKT, and (**C**) GPX4 in cortex of APP/PS1 mice (n = 3). (**D** to **G**) Quantification of proteins signals in Figure 4 normalized to GAPDH and expressed as fold of WT mice (n = 3). The data are means ± S.E.M. $^{\#}p < 0.05$, $^{\#\#}p < 0.01$, $^{\#\#\#}p < 0.001$ vs. WT mice, $^{*}p < 0.05$, $^{**}p < 0.01$, $^{***}p < 0.001$, vs. APP/PS1 mice.

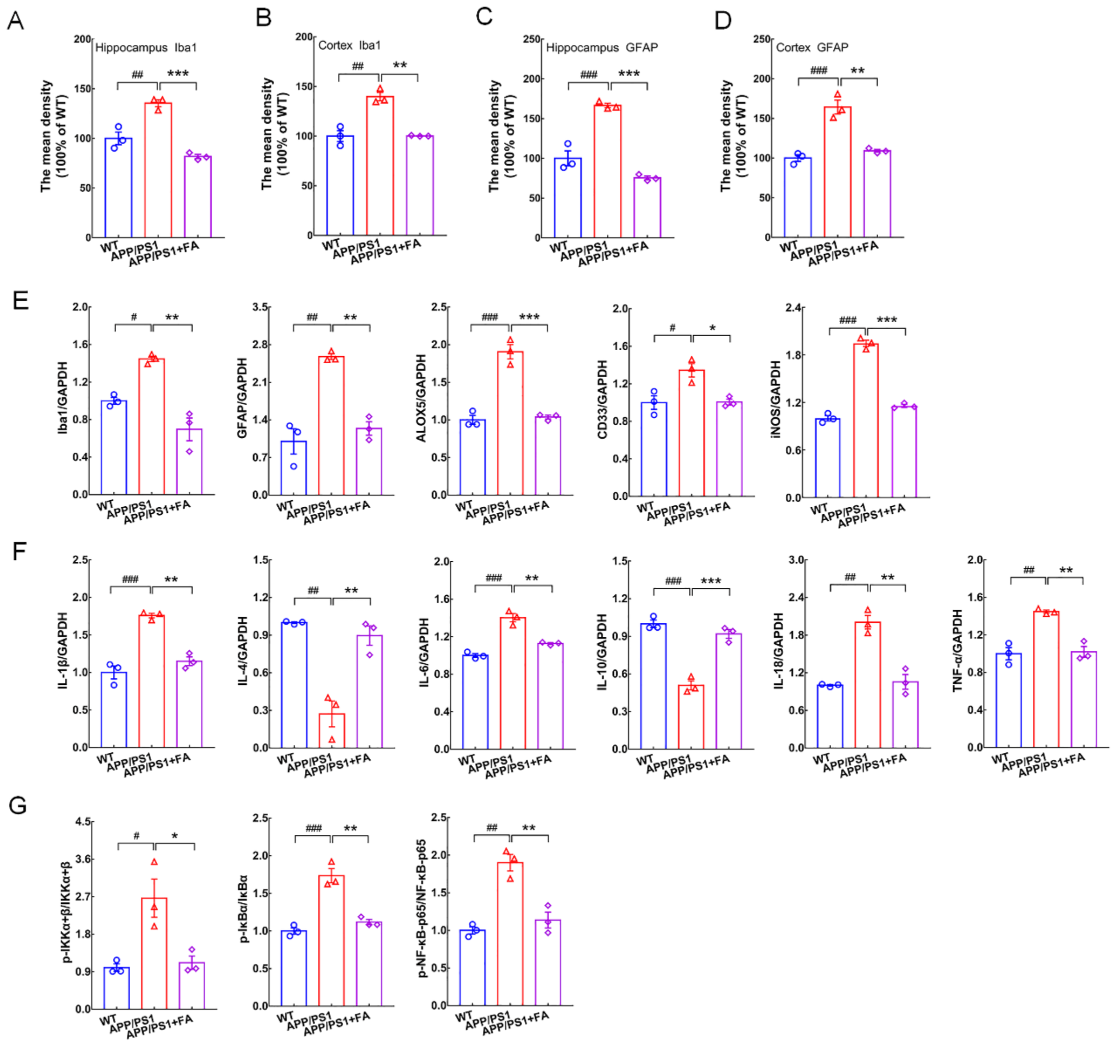

Figure S5. FA alleviates neuroinflammation in brains of APP/PS1 mice. (**A** to **D**) Quantification of the mean density of Iba1 and GFAP in hippocampus and cortex of APP/PS1 mice (n = 3). (**E** to **G**) Quantification of proteins signals in Figure 5 normalized to GAPDH and expressed as fold of WT mice (n = 3). The data are shown as means ± S.E.M. $^{\#}p < 0.05$, $^{\#\#}p < 0.01$, $^{\#\#\#}p < 0.001$ vs. WT mice, $^{*}p < 0.05$, $^{**}p < 0.01$, $^{***}p < 0.001$, vs. APP/PS1 mice.

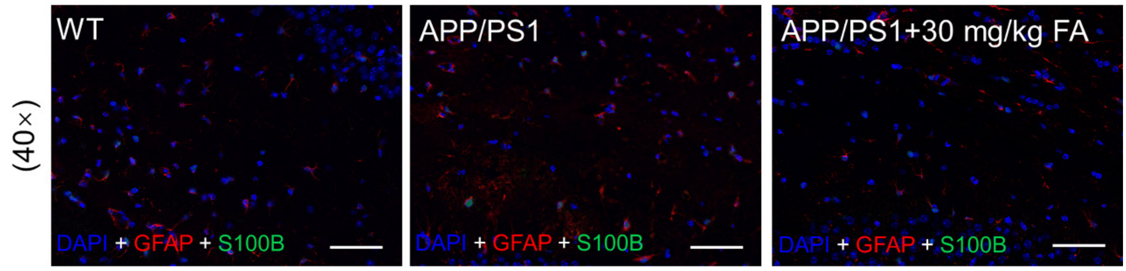

Figure S6. FA decreased expression levels of GFAP and S100B in the brain of APP/PS1 mice (scale bar: 50 μm) (n = 3).

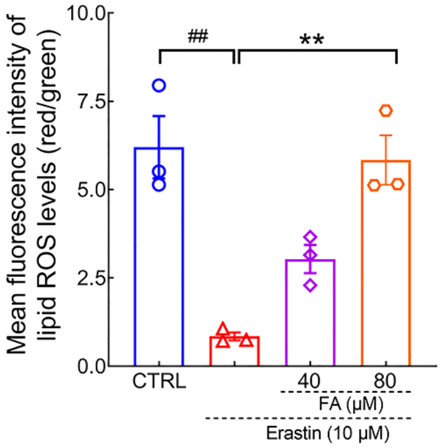

Figure S7. The quantification of the mean fluorescence intensity of lipid ROS levels (n = 3). The data are shown as means ± S.E.M. $^{##}p < 0.01$ vs. CTRL HT22 cells, $^{**}p < 0.01$ vs. erastin-exposed HT22 cells.

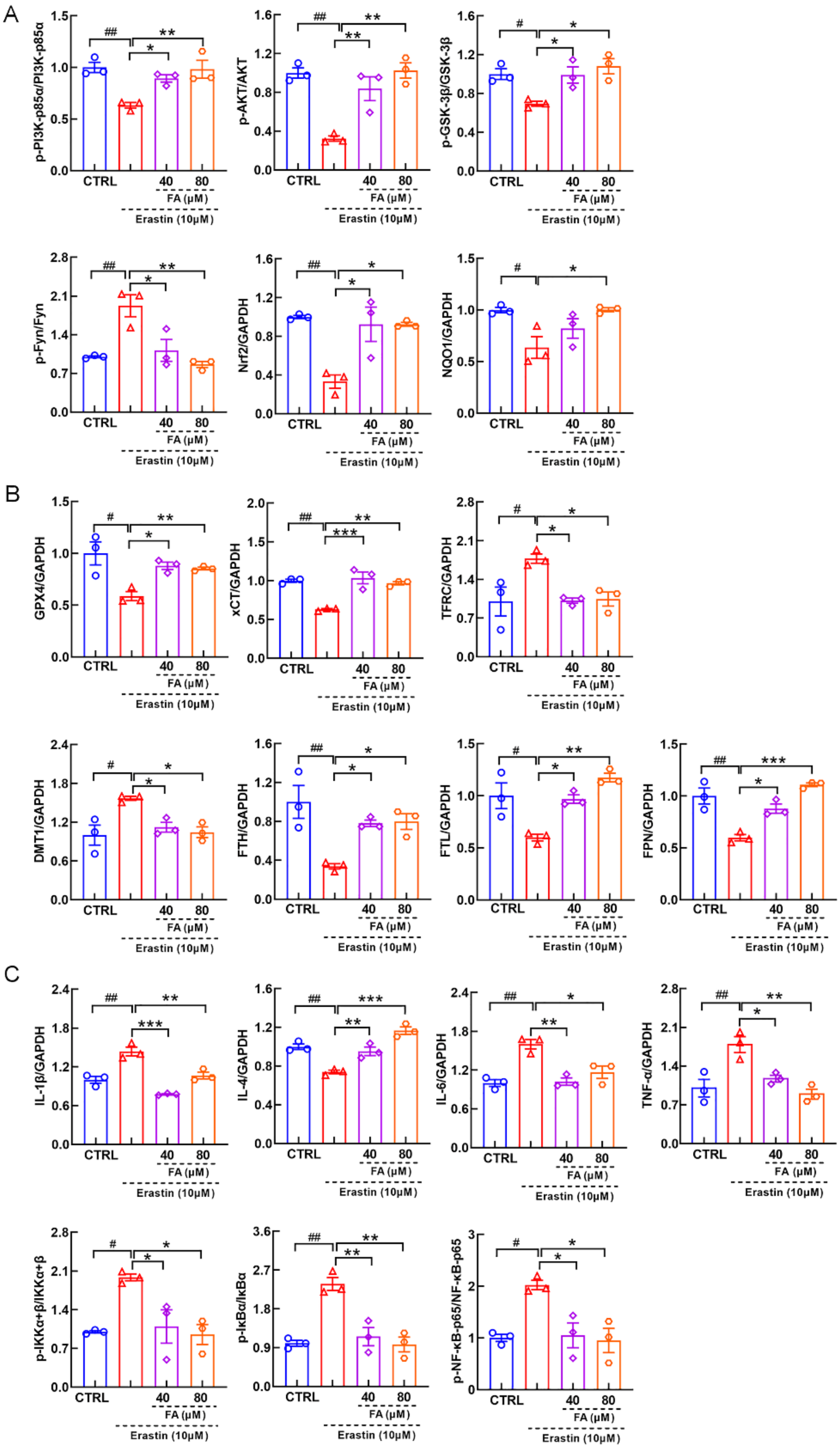

Figure S8. FA alleviates ferroptosis related inflammation in erastin-exposed HT22 cells. Quantification of proteins signals in Figure 6 normalized to GAPDH and expressed as fold of CTRL HT22 cells (n = 3). The data are shown as means ± S.E.M. $^{\#}p < 0.05$, $^{\#\#}p < 0.01$ vs. CTRL HT22 cells, $^{*}p < 0.05$, $^{**}p < 0.01$, $^{***}p < 0.001$ vs. erastin-exposed HT22 cells.

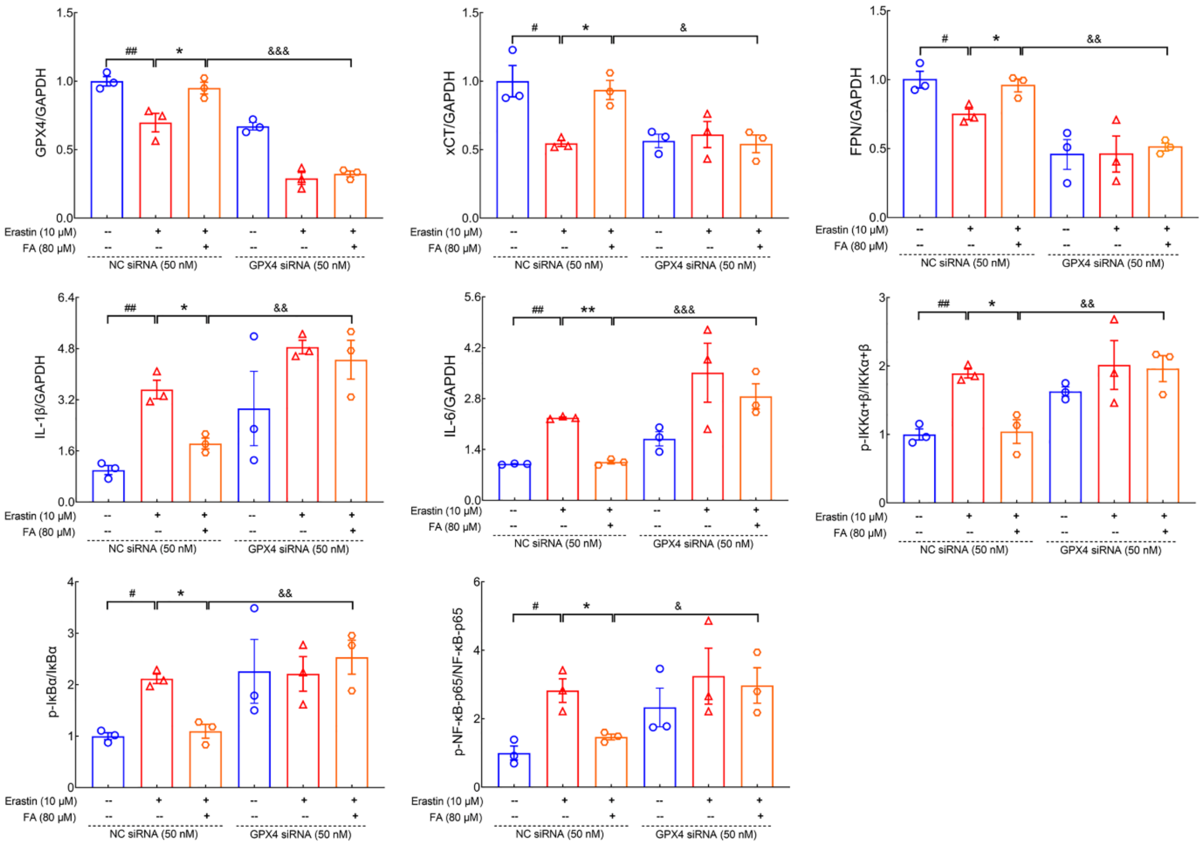

Figure S9. The involvement of GPX4 during FA-mediated anti-ferroptosis and anti-neuroinflammation. Quantification of proteins signals in Figure 7B normalized to GAPDH and expressed as fold of CTRL HT22 cells transfected with NC siRNA (n = 3). The data are shown as means ± S.E.M. $^{\#}p < 0.05$, $^{\#\#}p < 0.01$ vs. CTRL HT22 cells transfected with NC siRNA, $^{*}p < 0.05$, $^{**}p < 0.01$ vs. erastin-exposed HT22 cells transfected with NC siRNA, $^{\&}p < 0.05$, $^{\&\&}p < 0.01$, $^{\&\&\&}p < 0.001$ vs. FA-treated HT22 cells transfected with NC siRNA.

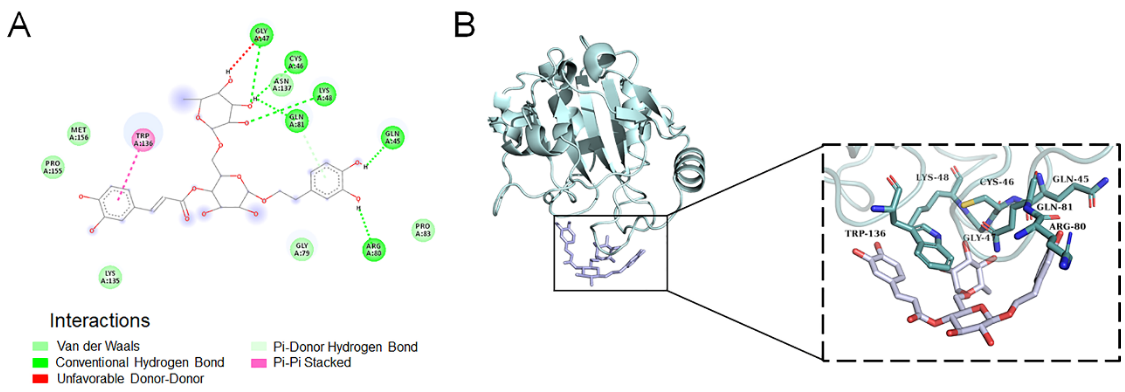

Figure S10. Active residues around FA binding to GPX4. (A) The interactions between FA and GPX4. **(B)** The binding poses of FA**.**

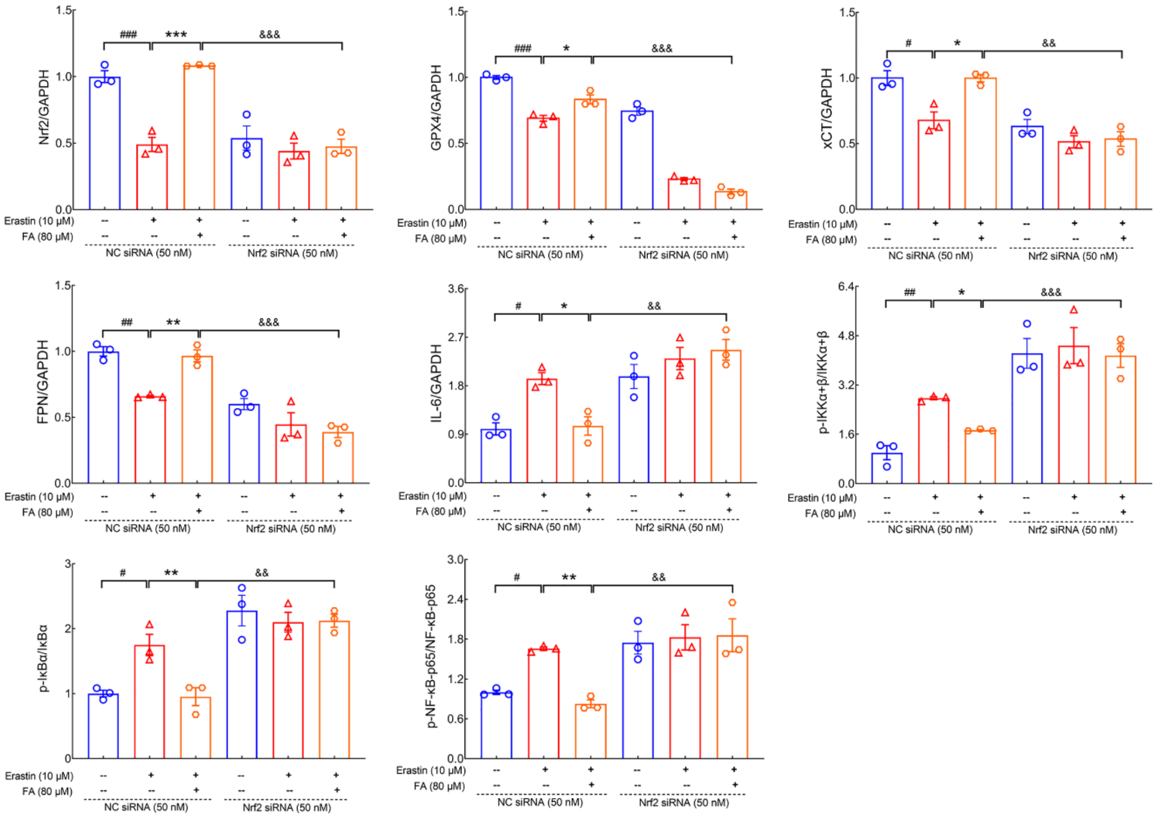

Figure S11. The involvement of Nrf2 during FA-mediated anti-ferroptosis and anti-neuroinflammation. Quantification of proteins signals in Figure 7D normalized to GAPDH and expressed as fold of CTRL HT22 cells transfected with NC siRNA (*n* = 3). The data are shown as means ± S.E.M. $^{\#}p < 0.05$, $^{\#\#}p < 0.01$, $^{\#\#\#}p < 0.001$ vs. CTRL HT22 cells transfected with NC siRNA, $^{*}p < 0.05$, $^{**}p < 0.01$, $^{***}p < 0.001$ vs. erastin-exposed HT22 cells transfected with NC siRNA, $^{\&\&}p < 0.01$, $^{\&\&\&}p < 0.001$ vs. FA-treated HT22 cells transfected with NC siRNA.

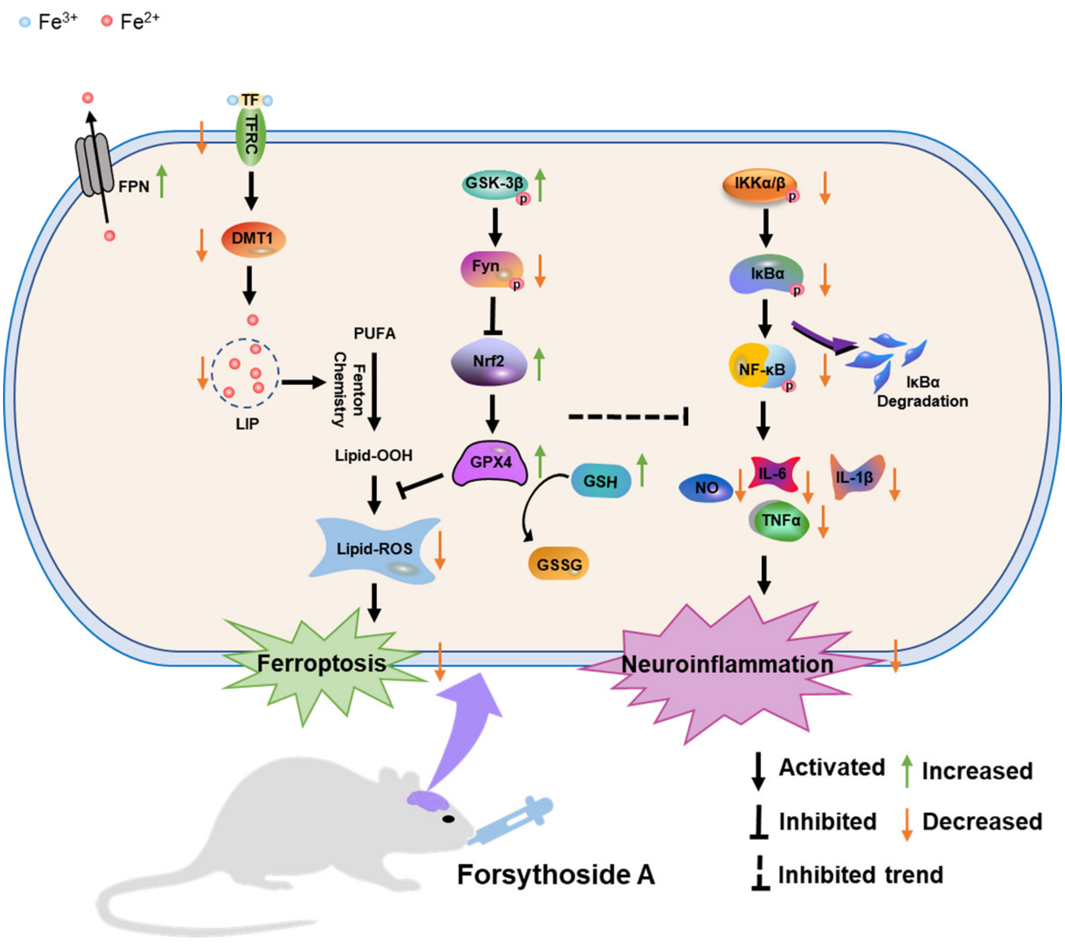

Figure S12. A diagram about the possible neuroprotective mechanisms of FA against AD-related ferroptosis and neuroinflammation via Nrf2/GPX4 axis.

References

[1] Wang CY, Cai XY, Hu WJ, Li ZP, Kong FG, Chen X, Wang D. Investigation of the neuroprotective effects of crocin via antioxidant activities in HT22 cells and in mice with Alzheimer's disease. Int J Mol Med. 2019;43(2):956-66.

[2] Wang CY, Cai XY, Wang RC, Zhai SY, Zhang YF, Hu WJ, Zhang YZ, Wang D. Neuroprotective effects of verbascoside against Alzheimer's disease via the relief of endoplasmic reticulum stress in A beta-exposed U251 cells and APP/PS1 mice. J Neuroinflamm. 2020;17(1).
